# Short-Term Wind Power Forecasting Using an HOA–VMD–CNN–iTransformer–LSTM Deep Learning Framework

**DOI:** 10.3390/s26217028

**Published:** 2026-11-06

**Authors:** Huanjun Chen, Yan Wang, Jingran Sheng, Xinfu Pang, Zedong Zheng

**Affiliations:** 1 Key Laboratory of Energy Saving and Controlling in Power System of Liaoning Province, Shenyang Institute of Engineering, Shenyang 110136, China; 12025101327@stu.sie.edu.cn (H.C.); shengjr@sie.edu.cn (J.S.); pangxf@sie.edu.cn (X.P.); 2 School of Computing and Mathematical Sciences, University of Leicester, Leicester LE1 7RH, UK; zz288@leicester.ac.uk

**Keywords:** hyperparameter optimization, iTransformer, LSTM, short-term wind power forecasting, variational mode decomposition

## Abstract

Reliable short-term wind power forecasts are difficult to obtain because power records are strongly non-stationary and hybrid networks are highly sensitive to parameter choices. This study proposes a Pearson–HOA–VMD–CNN–iTransformer–LSTM framework to address these challenges. Pearson coefficients are used to screen the candidate meteorological inputs. The hiking optimization algorithm (HOA) subsequently searches the mode number and penalty parameter of variational mode decomposition (VMD), together with the principal hyperparameters of the forecasting network. The optimized decomposition reduces sequence complexity, while a convolutional neural network (CNN) extracts local fluctuation patterns. iTransformer captures global variable dependencies using variate-wise self-attention, and long short-term memory (LSTM) models learn long-term temporal information. The framework was evaluated using four datasets containing 2976, 2880, 2976, and 2976 records, respectively, drawn from 2020 operational data from two Chinese wind farms with installed capacities of 99 and 200 MW. The sampling interval was 15 min, and the forecast horizons were 15, 30, and 45 min. Tests on four datasets show consistent gains over CNN–iTransformer–LSTM. Averaged across the datasets at the 15-min forecast horizon, RMSE, MAE, and MAPE decrease by 67.48%, 71.17%, and 49.66%, respectively, and mean R^2^ rises from 0.9672 to 0.9974.

## 1. Introduction

Continued growth in global energy demand has increased fossil fuel consumption and associated greenhouse gas emissions, further intensifying climate change. To accelerate the low-carbon transition of energy systems, many countries have established carbon-peaking and carbon-neutrality targets and expanded renewable energy deployment. Among renewable sources, wind energy is now widely integrated into contemporary power systems. Wind energy has also been investigated in hybrid photovoltaic–wind systems for hydrogen production [1]. However, its output varies with wind speed, wind direction, temperature, atmospheric pressure, and turbine operating conditions, resulting in substantial randomness, intermittency, and volatility. As grid-connected wind capacity increases, these characteristics complicate power balancing, reserve allocation, and scheduling decisions and may increase operational risks within the power system. Therefore, reliable wind power forecasting (WPF) helps optimize wind farm operation, improve grid scheduling, and facilitate wind energy integration. In this study, forecasts 15, 30, and 45 min ahead are intended to provide advance information for intra-hour wind farm scheduling, grid balancing, and reserve adjustment.

Short-term WPF maps recent meteorological measurements and power records to future wind farm generation using multivariate sequence modelling. Physical models generally rely on physical parameters, making their performance sensitive to parameter completeness and quality.

Traditional statistical models have limited capacity to represent rapid fluctuations and nonlinear dependencies in wind power records. By contrast, artificial intelligence models offer strong nonlinear fitting and feature learning capabilities and can integrate meteorological variables with historical wind power observations. Nevertheless, existing artificial intelligence-based forecasting approaches face three major challenges. First, a single architecture rarely captures local fluctuation patterns, global inter-variable dependencies, and long-term temporal information. Second, high volatility and non-stationarity in raw wind power sequences complicate model training and can impair forecasting performance. Third, decomposition methods and deep learning models involve numerous hyperparameters, making manual tuning difficult. An integrated framework combining feature selection, signal decomposition, multilevel feature learning, and intelligent parameter optimization is therefore needed to improve forecasting accuracy, stability, and adaptability.

### 1.1. Literature Review

Accurate WPF informs system dispatch, reserve planning, electricity market decisions, and renewable energy integration [2]. Four WPF categories are distinguished by their forecast scales: ultra-short-term, short-term, medium-term, and long-term prediction [3]. Ultra-short-term forecasting generally spans seconds to minutes and supports real-time grid regulation and wind farm control [4]. Short-term forecasting covers thirty minutes to six hours [5], whereas medium- and long-term forecasting serves maintenance scheduling, electricity trading, capacity expansion, and grid planning [6]. Improving forecasting accuracy can mitigate the adverse effects of wind power variability and uncertainty on power system operation [7].

Current WPF approaches fall into three main groups: physics-based, conventional statistical, and artificial intelligence methods. Physical methods estimate output from numerical weather predictions, terrain descriptions, turbine power curves, and operating data. Reference [8] combined physical constraints with data-driven learning to improve offshore wind forecasting. Reference [9] used supervisory control and data acquisition (SCADA) records alone to train a physics-aware residual ensemble for wind power forecasting. Although physical models provide a degree of interpretability, they require accurate meteorological inputs and detailed wind farm parameters. Their forecasting performance is therefore sensitive to numerical weather prediction errors, complex terrain, and variations in turbine operating conditions.

Conventional statistical models establish forecasting relationships by extracting temporal correlations from historical wind speed or wind power sequences. These techniques range from autoregressive moving average (ARMA) models and their integrated and seasonal extensions (ARIMA and SARIMA) to Gaussian process regression. Reference [10] employed ARMA for hourly wind speed forecasting and demonstrated its applicability to relatively stationary time series. Reference [11] combined ARIMA with an artificial neural network to forecast wind speed in different regions. Reference [12] reviewed uncertainty sources in WPF and showed that both meteorological input and model errors affect forecasting reliability. Reference [13] addressed probabilistic WPF using a selectively constructed ensemble of finite mixture Gaussian process regression models. Although these techniques are efficient, their ability to characterize abrupt nonlinear variations in wind power generation is limited.

Compared with conventional statistical methods, artificial intelligence models provide stronger nonlinear fitting and autonomous feature learning capabilities. Reference [14] combined CNN and LSTM for probabilistic WPF. Reference [15] optimized LSTM using the attraction–repulsion optimization algorithm. References [16,17] developed CNN–BiLSTM–Attention and Res–ConvLSTM–Attention models, respectively, to integrate local and temporal features. Reference [18] combined temporal convolutional network (TCN) and LSTM in a parallel architecture and applied Savitzky–Golay filtering to smooth the input signal. Nevertheless, CNNs primarily capture local patterns, whereas recurrent networks may lose long-range information when processing long input sequences.

Transformer-based models have therefore been introduced to strengthen global-dependency modeling. Reference [19] proposed Powerformer to capture long-range temporal relationships in wind power sequences. Reference [20] integrated empirical mode decomposition, causal convolution, and Transformer for ultra-short-term WPF. References [21,22] combined Transformer with BiGRU and LSTM, respectively, demonstrating the complementarity between attention-based global modeling and recurrent temporal learning. Reference [23] used temporal collaborative attention to capture variable relationships and fuse short- and long-term information. However, conventional Transformers generally combine all variables observed at the same time step into a token and apply self-attention along the temporal dimension. Consequently, they focus mainly on relationships between temporal positions.

Reference [24] proposed iTransformer, which represents the complete historical sequence of each variable as an independent variate token and applies self-attention across the variate dimension. Related Transformer extensions include the graph-based GNNCrossformer [25], the sparse attention Pt-Transformer [26], prompt-enhanced foundation time-series models [27], and Transformer models combined with transfer learning [28]. Reference [29] proposed FSDIformer, which separates wind power sequences into trend and periodic components and models them using downsampling interaction and frequency domain sparse attention, respectively. Although these methods extend Transformer-based WPF, they may require spatial topology, source domain data, or considerable computational resources. Transformer-based models do not universally outperform simpler forecasting approaches. Reference [30] showed that simple linear models outperformed the evaluated Transformer models on several long-term forecasting benchmarks. A relatively concise architecture that simultaneously models local patterns, inter-variable dependencies, and continuous temporal dynamics is therefore still required.

Random variations and changing statistical properties make raw wind power records difficult to learn directly. Decomposition-based methods address this problem by separating the signal into frequency-specific modes, predicting them independently, and combining their outputs. Reference [31] combined wavelet decomposition with LSTM, whereas Reference [32] integrated multivariate signal decomposition with variable selection. References [33,34] developed two-layer and CEEMDAN–EWT decomposition frameworks, respectively. Reference [35] integrated CEEMDAN–VMD decomposition, KPCA, intelligent optimization, and BiLSTM with error correction for multi-step forecasting. These approaches decompose the original wind power series into simpler components.

Reference [36] developed variational mode decomposition (VMD), which represents a non-stationary signal as several band modes. Compared with empirical mode decomposition methods, VMD generally provides stronger frequency separation and resistance to mode mixing. Reference [37] used the butterfly optimization algorithm to determine the VMD mode number k, penalty factor α, and LSTM hyperparameters. Reference [38] determined the mode number using sparse prior knowledge and combined VMD with LASSO–BiGRU. Reference [39] developed a VMD–CNN–GRU model for local and temporal feature extraction. Reference [40] integrated CEEMDAN, VMD, feature enhancement, and Transformer–GRU, whereas Reference [41] combined wake effect modeling, VMD, and deep learning. Nevertheless, an inappropriate k may cause under-decomposition or over-decomposition, while an unsuitable α can impair modal bandwidth and frequency separation.

Intelligent optimization algorithms have consequently been used to configure both decomposition and parameters of forecasting models. Reference [42] integrated improved preprocessing with an entropy-weighted GRU quantile regression network. Reference [43] combined multivariate decomposition, RIME optimization, and BiLSTM. Reference [44] employed RIME to optimize both VMD parameters and TCN hyperparameters, whereas Reference [45] combined two-layer adaptive decomposition with multi-objective optimization. Reference [46] therefore employed an improved snake optimization algorithm to tune LSTM hyperparameters. Reference [47] compared Scikit-opt, Optuna, and Hyperopt for CNN and LSTM tuning, and Reference [48] used an improved coot optimization algorithm to optimize five internal hyperparameters of an LSTM–TCN–Self-Attention model.

Reference [49] proposed the hiking optimization algorithm (HOA), which simulates hikers’ movement to coordinate global exploration and local exploitation. Reference [50] developed an improved HOA for renewable energy wind speed prediction, and Reference [51] subsequently employed HOA to optimize VMD parameters for short-term WPF.

### 1.2. Research Gaps and Contributions

Recent studies have increasingly combined input screening and signal decomposition with intelligent parameter search and deep neural networks for renewable energy forecasting. These methods have improved the prediction accuracy. However, several limitations remain. First, although signal decomposition reduces the complexity of wind power sequences, accurate component forecasting still requires effective integration of local fluctuations, inter-variable relationships, and temporal dependencies. Second, the decomposed components differ in frequency and variability, making a shared network configuration potentially unsuitable for all components. Further investigation is therefore needed into hybrid feature extraction and component-specific hyperparameter optimization to improve decomposition-based wind power forecasting.

In this study, the proposed framework screens the meteorological inputs using Pearson correlation analysis and applies HOA-optimized VMD to separate the raw wind power signal into less complex modes. Then CNN is initially employed to extract local fluctuation patterns from the decomposed components and selected meteorological variables. However, CNN has limited capability to capture global dependencies among variables. To address this limitation, we introduced iTransformer to capture global dependencies among multiple variables. LSTM is incorporated to further learn the long-term information from wind power data. During model training, HOA is further employed to optimize the key hyperparameters of the CNN–iTransformer–LSTM network, thereby reducing the subjectivity of manual parameter configuration and improving the adaptability of the forecasting model to complex wind power data. Accordingly, this study proposes a hierarchical Pearson–HOA–VMD–CNN–iTransformer–LSTM model for short-term WPF. The study contributes in three respects:

(1) A hierarchical HOA-based parameter optimization strategy is developed for both data decomposition and deep forecasting. HOA adaptively determines the mode number and penalty factor of VMD and further optimizes the key hyperparameters of the CNN–iTransformer–LSTM network. This strategy coordinates the preprocessing and forecasting stages while reducing the dependence on manually configured parameters.
(2) We proposed an iTransformer-centered CNN–iTransformer–LSTM architecture. CNN extracts local fluctuation patterns from the wind power data. iTransformer then applies variate-wise self-attention to capture global dependencies among multiple variables, while LSTM further learns long-term information.
(3) The framework is assessed using four operational datasets obtained from two wind farms of different capacities. Comparisons with single, hybrid, decomposition-based, and alternative optimization models, together with one- to three-step tests, evaluate accuracy, stability, and cross-dataset generalization.

The remaining parts of this study are structured as follows. Section 2 defines the forecasting problem and outlines the proposed strategy. Section 3 presents the constituent algorithms, forecasting network, and HOA-based optimization procedure. Section 4 specifies the data preprocessing procedures and performance metrics. Section 5 reports and discusses the experiments, and Section 6 concludes the study.

## 2. Problem Description and Strategy

### 2.1. Problem Description

Short-term WPF is formulated as a multivariate time-series problem. Each input combines recent meteorological measurements with historical power output, while the response is wind farm output at target time *t*. The *i*-th input batch is defined as follows:

(1) $$X_{i}=\left({\vec{x}}_{1},{\vec{x}}_{2},{\vec{x}}_{3},\ldots ,{\vec{x}}_{n}\right)$$

where *X_i_* represents the *i*-th input samples and ${\vec{x}}_{n}$ denotes the *n*-th input sample in this batch. The input sample ${\vec{x}}_{n}$ can be expressed as follows:

(2) $${\vec{x}}_{n}=\left(F_{t-1}^{1},F_{t-1}^{2},F_{t-1}^{3},F_{t-1}^{4},F_{t-1}^{5},P_{t-1},P_{t-2},P_{t-3},P_{t-4},P_{t-5}\right),t\in \left(1,n\right)$$

where $F_{t-1}^{i},i\in \left(1,5\right)$ denotes the *i*-th meteorological feature at moment *t −* 1 and $P_{t}$ denotes the wind power at moment t.

In Equation (2), $F_{t-1}^{1},...,F_{t-5}^{5}$ denote the five meteorological variables retained through PCC screening. Together with the five lagged power values $P_{t-1},...,P_{t-5}$, they form a 10-element input vector.

### 2.2. Strategy Structure

**Figure 1:**
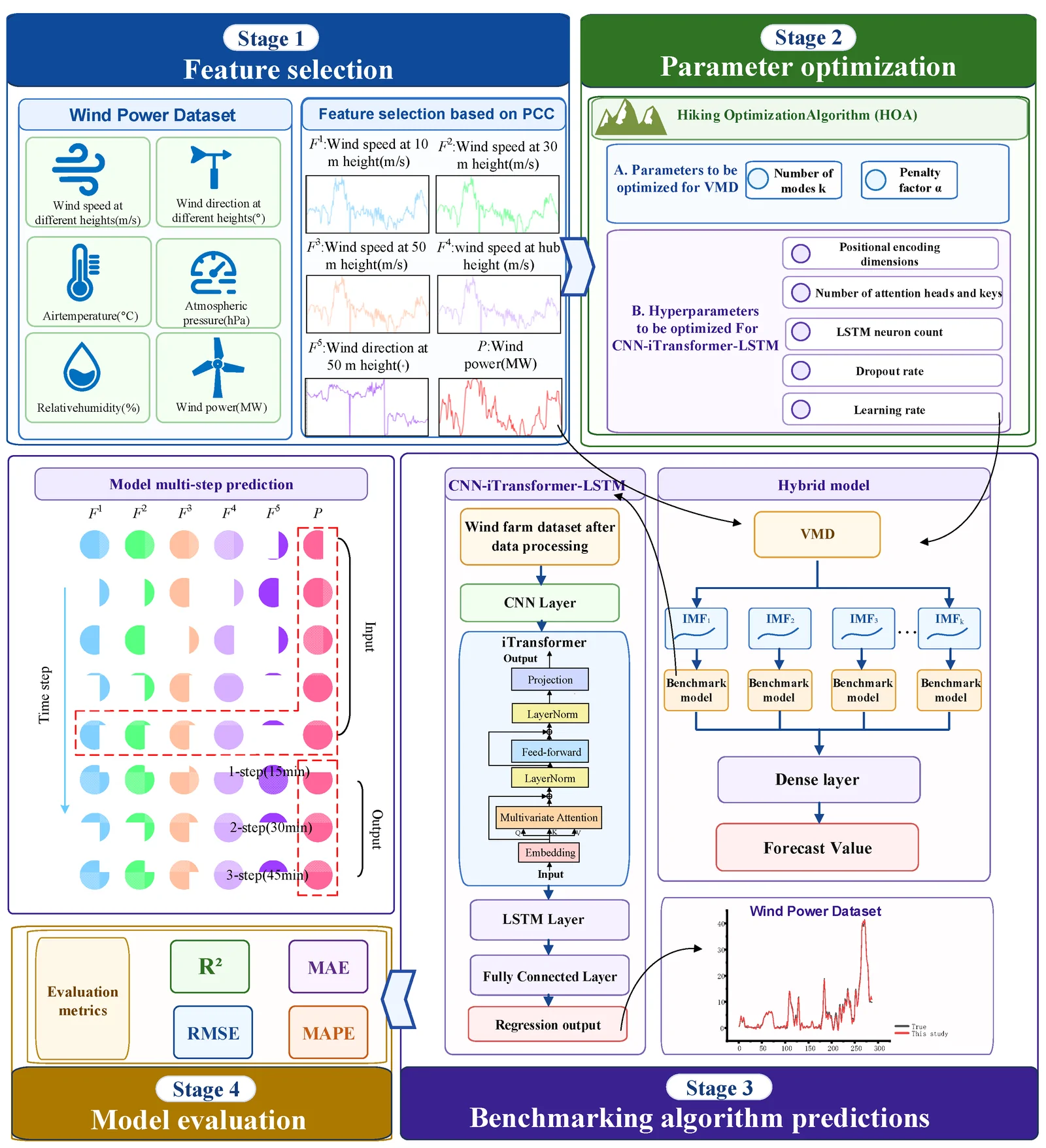
Schematic of research on short-term wind power forecasting.

Figure 1 summarizes the four stages of the proposed strategy. First, Pearson coefficients identify the meteorological variables most closely associated with power, thereby limiting redundant input dimensions. Second, HOA searches the VMD settings and the principal CNN–iTransformer–LSTM hyperparameters. Third, the optimized VMD separates the observed power sequence and paired with the selected meteorological inputs and forecast using the optimized network. Finally, the component forecasts are summed, returned to the original scale, and assessed.

The complete forecasting workflow comprises the following seven steps:

Step 1: Preprocess the data. Detect missing observations, estimate them using cubic spline interpolation, and apply Z-score standardization to every retained variable.
Step 2: Select informative inputs. Compute the Pearson correlation coefficient (PCC) between each meteorological variable and power, retain the relevant variables, and discard weak or redundant inputs.
Step 3: Optimize and apply VMD. HOA searches the mode number K and penalty factor α using the defined decomposition fitness function. VMD with the selected settings then separates the original power sequence into K modes.
Step 4: Configure the forecasting network. A second HOA search determines the position-encoding dimension, number of attention heads, number of LSTM neurons, number of attention keys, learning rate, and dropout rate of the CNN–iTransformer–LSTM model.
Step 5: Forecast individual components. For each decomposed component, CNN encodes short-range variations, iTransformer relates variables using variate-wise attention, and LSTM models their sequential evolution.
Step 6: Reconstruct the power forecast. Sum the predicted modes and residual. Then, reverse the standardization to express the result on the original power scale.
Step 7: Evaluate performance. Calculate MAE, RMSE, MAPE, and R^2^ from the reconstructed forecasts and observations.

## 3. CNN–iTransformer–LSTM Prediction Method Based on HOA Optimization

### 3.1. Principles and Design of Related Algorithms

#### 3.1.1. Variational Mode Decomposition

Variational mode decomposition (VMD) formulates the decomposition of a non-stationary signal as a variational optimization problem. It represents the input signal using several band-limited modes centered at different frequencies. Wind power records contain slow trends, rapid fluctuations, and noise because generation varies with meteorological and operating conditions. Learning these mixed frequency dynamics directly may complicate model training. Therefore, VMD is applied to the original wind power series before forecasting, producing modal components with simpler frequency characteristics.

Let $f\left(t\right)$ denote the original wind power signal. VMD estimates *K* modes $u_{k}\left(t\right)$ and their corresponding center frequencies $\omega_{k}$ by minimizing the sum of the modal bandwidths under a reconstruction constraint. The resulting constrained optimization problem is defined as follows:

(3) $$\left\{\begin{array}{c} \underset{\left\{u_{k},\omega_{k}\right\}}{\min}\left\{\sum_{k=1}^{K}{\left\|\frac{\partial }{\partial t}\left[\left(\delta \left(t\right)+\frac{j}{\pi t}\right)*u_{k}\left(t\right)\right]e^{-j\omega_{k}t}\right\|}_{2}^{2}\right\}\\ s.t.\sum_{k=1}^{K}u_{k}\left(t\right)=f\left(t\right) \end{array}\right.$$

where $u_{k}\left(t\right)$ is the *k*-th modal component and $\omega_{k}$ is the center frequency. In addition, $\delta \left(t\right)$, j, and ∗ denote the Dirac distribution, imaginary unit, and convolution operator, respectively.

To obtain a numerical solution, the reconstruction constraint is incorporated into the objective function using the quadratic penalty parameter α and Lagrange multiplier $\lambda \left(t\right)$. The corresponding augmented Lagrangian is written as follows:

(4) $$\begin{array}{l} L\left(\left\{u_{k}\right\},\left\{\omega_{k}\right\},\lambda \right)=\\ \alpha \sum_{k=1}^{K}{\left\|\frac{\partial }{\partial t}\left[\left(\delta \left(t\right)+\frac{j}{\pi t}\right)*u_{k}\left(t\right)e^{-j\omega_{k}t}\right]\right\|}_{2}^{2}+{\left\|f\left(t\right)-\sum_{k=1}^{K}u_{k}\left(t\right)\right\|}_{2}^{2}+\langle \lambda \left(t\right),f\left(t\right)-\sum_{k=1}^{K}u_{k}\left(t\right)\rangle \end{array}$$

The optimal solution of the augmented Lagrangian function is obtained by alternately updating the modal components, center frequencies, and Lagrange multiplier using the alternating direction multiplier method. The update formula of the modal component is given as follows:

(5) $${\hat{u}}_{k}^{n+1}\left(\omega \right)=\frac{\hat{f}\left(\omega \right)-\sum_{i<k}{\hat{u}}_{i}^{n+1}\left(\omega \right)-\sum_{i>k}{\hat{u}}_{i}^{n}\left(\omega \right)+\frac{{\hat{\lambda }}^{n}\left(\omega \right)}{2}}{1+2\alpha {\left(\omega -\omega_{k}^{n}\right)}^{2}}$$

The update formula of the center frequency is expressed as follows:

(6) $$\omega_{k}^{n+1}=\frac{\int_{0}^{\infty }\omega {\left|{\hat{u}}_{k}^{n+1}\left(\omega \right)\right|}^{2}d\omega }{\int_{0}^{\infty }{\left|{\hat{u}}_{k}^{n+1}\left(\omega \right)\right|}^{2}d\omega }$$

The Lagrange multiplier is updated as follows:

(7) $${\hat{\lambda }}^{n+1}\left(\omega \right)={\hat{\lambda }}^{n}\left(\omega \right)+\tau \left(\hat{f}\left(\omega \right)-\sum_{k=1}^{K}{\hat{u}}_{k}^{n+1}\left(\omega \right)\right)$$

When the convergence condition is satisfied, the iteration is terminated, and the modal components are obtained:

(8) $$\sum_{k=1}^{K}\frac{{\left\|u_{k}^{n+1}-u_{k}^{n}\right\|}_{2}^{2}}{{\left\|u_{k}^{n}\right\|}_{2}^{2}}<\varepsilon$$

where ${\hat{u}}_{k}$, $\hat{f}$, and $\hat{\lambda }$ denote the Fourier transforms of $u_{k}$, f, and λ, respectively. In addition, τ represents the update parameter, *n* denotes the number of iterations, and ε denotes the convergence error.

Decomposition quality depends primarily on K and α. Too few modes leave dissimilar frequencies entangled, whereas too many create redundant components. The penalty controls modal bandwidth and thus the degree of frequency separation. Because fixed empirical settings may not suit all data, an optimization algorithm is used to select K and α adaptively.

#### 3.1.2. Convolutional Neural Network

A convolutional neural network (CNN) is a fundamental deep learning architecture that is widely employed for feature extraction and pattern recognition [52]. In this study, CNN is introduced to extract local fluctuation features from wind power sequences and meteorological variables. The original input sequence usually contains local nonlinear variations and short-term fluctuation patterns. CNN can effectively capture these local characteristics using convolution operations.

**Figure 2:**
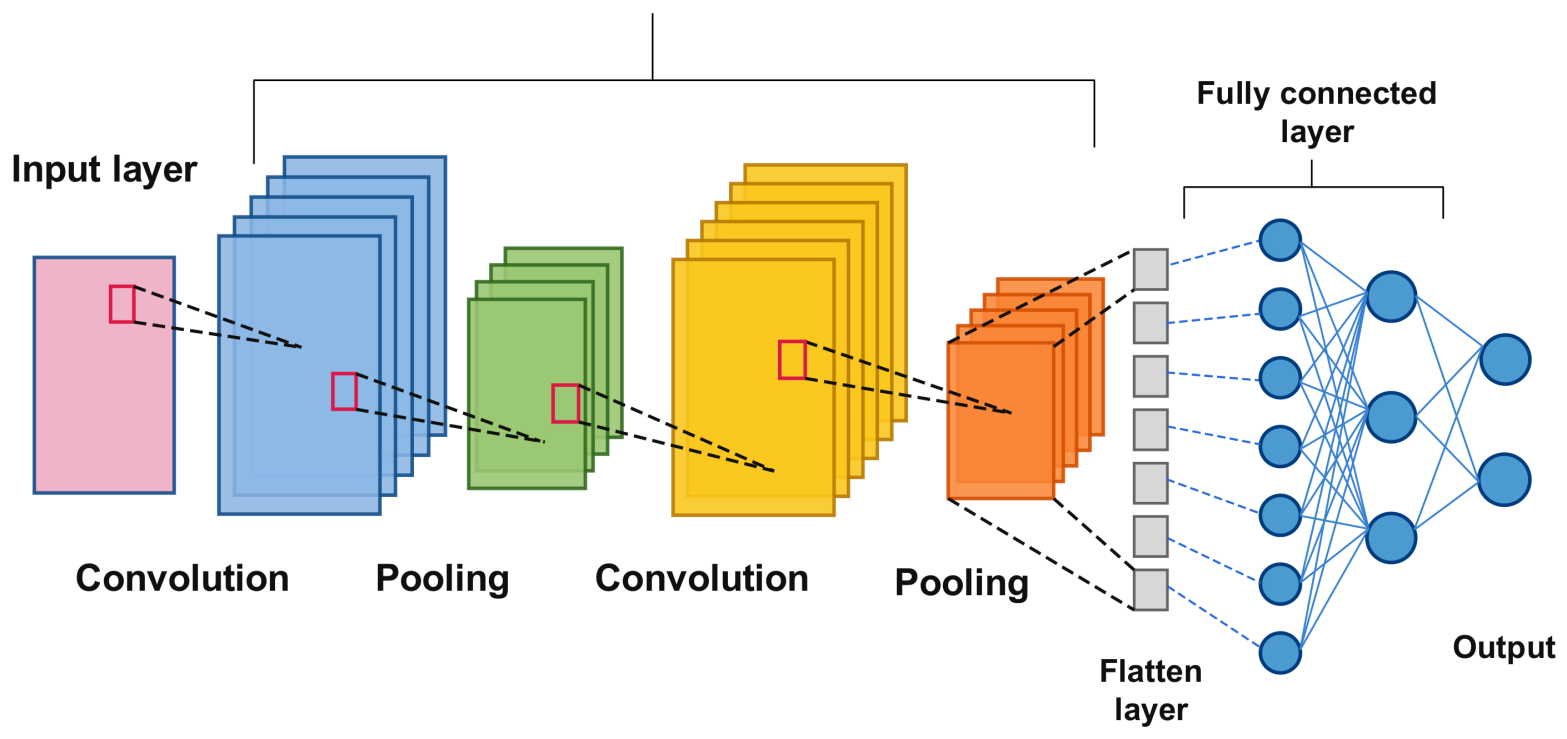
Structure of a convolutional neural network.

Figure 2 depicts the CNN architecture, comprising input, convolutional, pooling, flattening, fully connected, and output layers. In each convolutional layer, kernels scan the sequence along the time axis to identify local patterns. The resulting feature map is calculated as follows:

(9) $$H_{c}^{l}=\sigma \left(W_{c}^{l}*H_{c}^{l-1}+b_{c}^{l}\right)$$

where $H_{c}^{0}=X$ represents the input feature sequence; $W_{c}^{l}$ and $b_{c}^{l}$ denote the convolution kernel and bias term of the *l*-th convolutional layer, respectively; ∗ represents the convolution operation; $\sigma \left(\cdot \right)$ denotes the nonlinear mapping function; and $H_{c}^{l}$ is the output feature map of the *l*-th convolutional layer.

After convolution, pooling condenses the convolutional feature maps while preserving their strongest local responses, reducing redundant information. The pooled maps are then flattened into a vector and passed through a fully connected layer to produce the output representation.

#### 3.1.3. iTransformer

Wind farm power output depends on multiple meteorological factors and their nonlinear interactions over time. Although the traditional Transformer has been widely used for time-series forecasting, it usually regards the multivariate observations at the same timestamp as a temporal token and applies the attention mechanism along the temporal dimension [53]. This structure may weaken the modelling of multivariate correlations because different variables recorded at the same time often have distinct physical meanings and statistical distributions. Therefore, a more suitable structure is required to capture the correlations among meteorological variables and wind power output.

**Figure 3:**
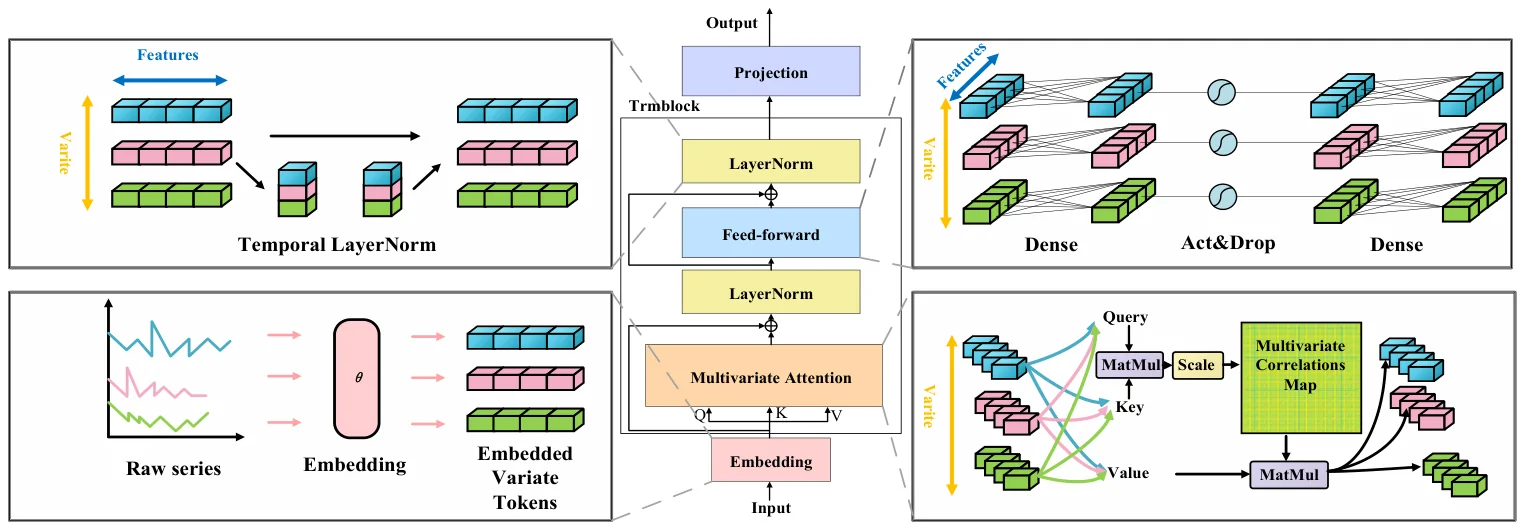
Structure of iTransformer.

In this study, iTransformer is employed to model the global dependency relationships among multiple variables, as depicted in Figure 3. Unlike a conventional Transformer, iTransformer embeds the complete historical sequence of each variable as a separate variate token. Self-attention then models interactions among these tokens, while the feed-forward network transforms each variable’s representation. It can incorporate relationships among meteorological variables and wind power output.

The historical multivariate time-series input is given as follows:

(10) $$X=\left\{x_{1},x_{2},...,x_{T}\right\}\in {\mathbb{R}}^{T\times N}$$

where *T* denotes the length of the lookback window and *N* represents the number of variables. In the iTransformer structure, $X_{:,n}$ denotes the complete historical sequence of the *n*-th variable. Each variable sequence is embedded independently into a variate token, which can be expressed as follows:

(11) $$h_{n}^{0}=Embedding\left(X_{:,n}\right)$$

where $h_{n}^{0}$ denotes the embedded representation of the n-th variable. The embedded token set is denoted as follows:

(12) $$H^{0}=\left\{h_{1}^{0},h_{2}^{0},...,h_{N}^{0}\right\}\in {\mathbb{R}}^{N\times D}$$

where D represents the dimension of the embedded variate token. The obtained variate tokens are then fed into stacked Transformer blocks:

(13) $$H^{l+1}=TrmBlock(H^{l}),l=0,1,...,L-1$$

where *L* denotes the number of iTransformer blocks. In each block, the self-attention module is applied to the variate token dimension to model the correlations among different variables. The query, key, and value matrices are obtained using linear projection:

(14) $$Q=HW_{Q},K=HW_{K},V=HW_{V}$$

where $W_{Q}$, $W_{K}$, and $W_{V}$ are trainable weight matrices. The attention score matrix is calculated as follows:

(15) $$A=softmax\left(\frac{QK^{T}}{\sqrt{d_{k}}}\right)$$

The weighted representation is obtained as follows:

(16) Z=AV

where $d_{k}$ is the projected dimension of the key vector. Since each variate token represents the complete historical sequence of one variable, the attention score matrix can reflect the correlation strength between different variables. Highly correlated variables are assigned larger attention weights, thereby enhancing the interaction of useful information among meteorological features and wind power output.

Finally, the future wind power sequence is generated using the projection layer:

(17) $${\hat{Y}}_{:,n}=Projection\left(h_{n}^{L}\right)$$

where $h_{n}^{L}$ represents the output representation of the n-th variable after L iTransformer blocks and $Y_{:,n}$ denotes the predicted future sequence. Using this inverted modelling strategy, iTransformer can effectively capture multivariate correlations and learn series-level representations, which provides strong global dependency modelling capability for wind power forecasting model.

#### 3.1.4. Long Short-Term Memory

LSTM regulates the retention and updating of information using its forget, input, and output gates, helping preserve dependencies across a sequence [54]. Following iTransformer, the LSTM uses gated state updates to retain relevant history and model temporal dependencies in wind power data.

At time step t, the forget gate determines the proportion of the previous cell state $C_{t-1}$ that is carried forward:

(18) $$f_{t}=\sigma \left(W_{f}\left[h_{t-1},x_{t}\right]+b_{f}\right)$$

The input gate assigns weights to new information derived from the current input $x_{t}$ and previous hidden state $h_{t-1}$:

(19) $$i_{t}=\sigma \left(W_{i}\left[h_{t-1},x_{t}\right]+b_{i}\right)$$

Meanwhile, a candidate memory vector is generated from the current input and preceding hidden representation:

(20) $${\tilde{C}}_{t}=tanh\left(W_{C}\left[h_{t-1},x_{t}\right]+b_{C}\right)$$

The cell state is updated by combining the retained historical memory with the gated candidate information:

(21) $$C_{t}=f_{t}⊙C_{t-1}+i_{t}⊙{\tilde{C}}_{t}$$

The output gate determines which part of the updated memory contributes to the hidden representation:

(22) $$o_{t}=\sigma \left(W_{o}\left[h_{t-1},x_{t}\right]+b_{o}\right)$$

The hidden state at the current time step is subsequently calculated as follows:

(23) $$h_{t}={ο}_{t}⊙\tanh\left(C_{t}\right)$$

**Figure 4:**
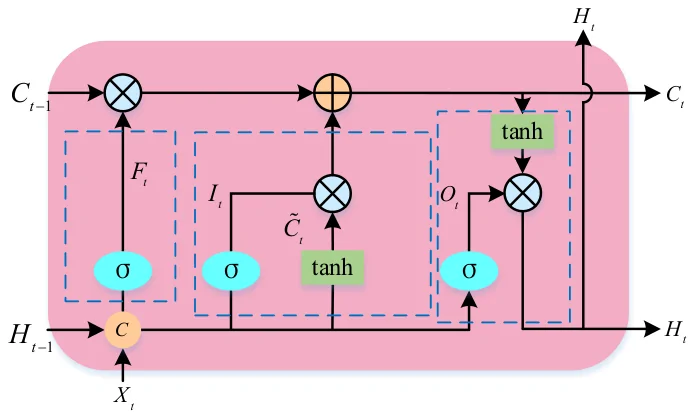
Structure of LSTM.

Finally, the hidden representation is mapped to the forecasting output using the prediction layer:

(24) $${\hat{y}}_{t}=f\left(W_{p}h_{t}+b_{p}\right)$$

where $x_{t}$ and $h_{t-1}$ denote the current input and previous hidden state, respectively. $C_{t}$ and $C_{t-1}$ represent the preceding and updated cell states. The terms $W_{f}$, $W_{i}$, $W_{c}$, $W_{o}$, and $W_{p}$ are trainable weight matrices, while $b_{f}$, $b_{i}$, $b_{c}$, $b_{o}$, and $b_{p}$ are their corresponding biases. $\sigma \left(\cdot \right)$ denotes the sigmoid function, $\tanh\left(\cdot \right)$ is the hyperbolic tangent function, and ⊙ represents element-wise multiplication. LSTM is shown in Figure 4.

### 3.2. CNN–iTransformer–LSTM Wind Power Forecast Model

#### 3.2.1. Description of Input Data

**Figure 5:**
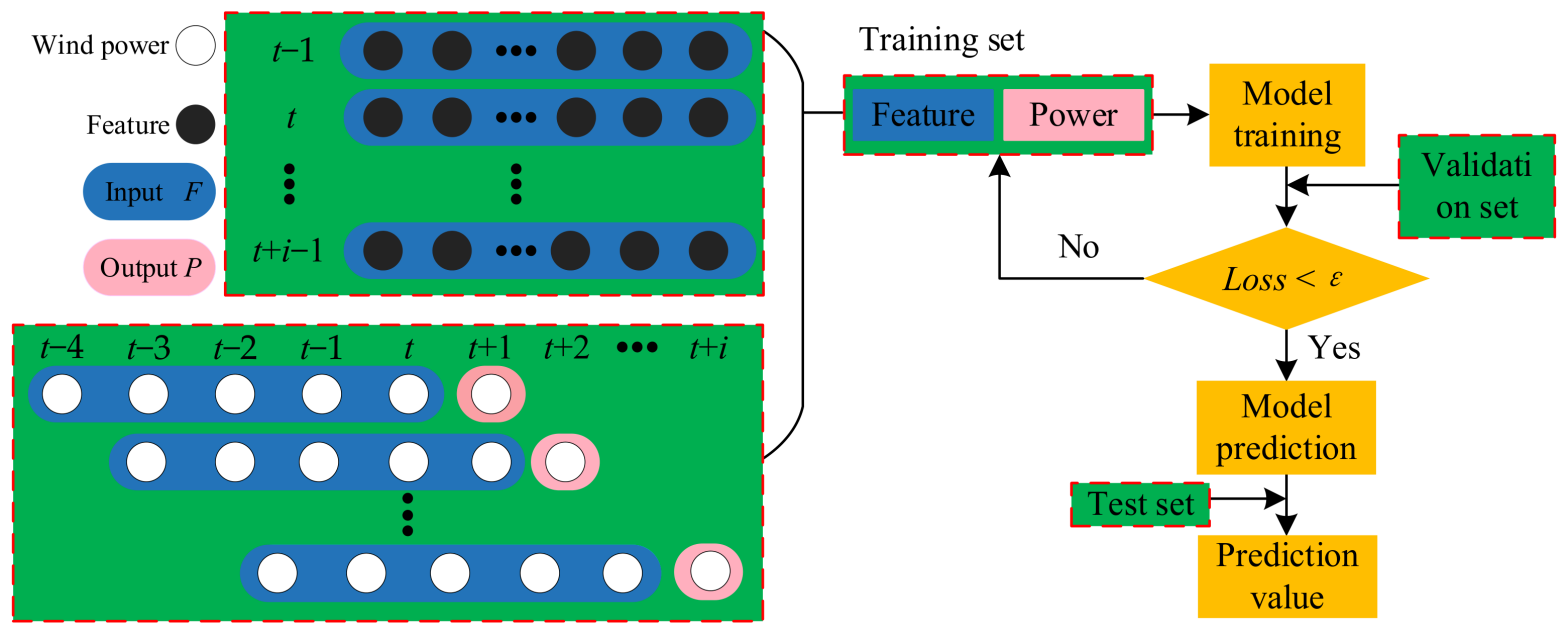
Structure of input data.

The historical data include wind speed and direction measured at multiple heights, as well as temperature, atmospheric pressure, relative humidity, and wind power. To predict output at time *t*, each input segment combines meteorological observations at *t −* 1 with power values from *t −* 5 to *t −* 1. Samples are spaced by 15 min, and successive segments are generated by advancing the window by one sampling interval. In the configuration shown in Figure 5, the most recent meteorological observations and five historical wind power values are concatenated into an input vector.

#### 3.2.2. Framework for the Forecasting Model

**Figure 6:**
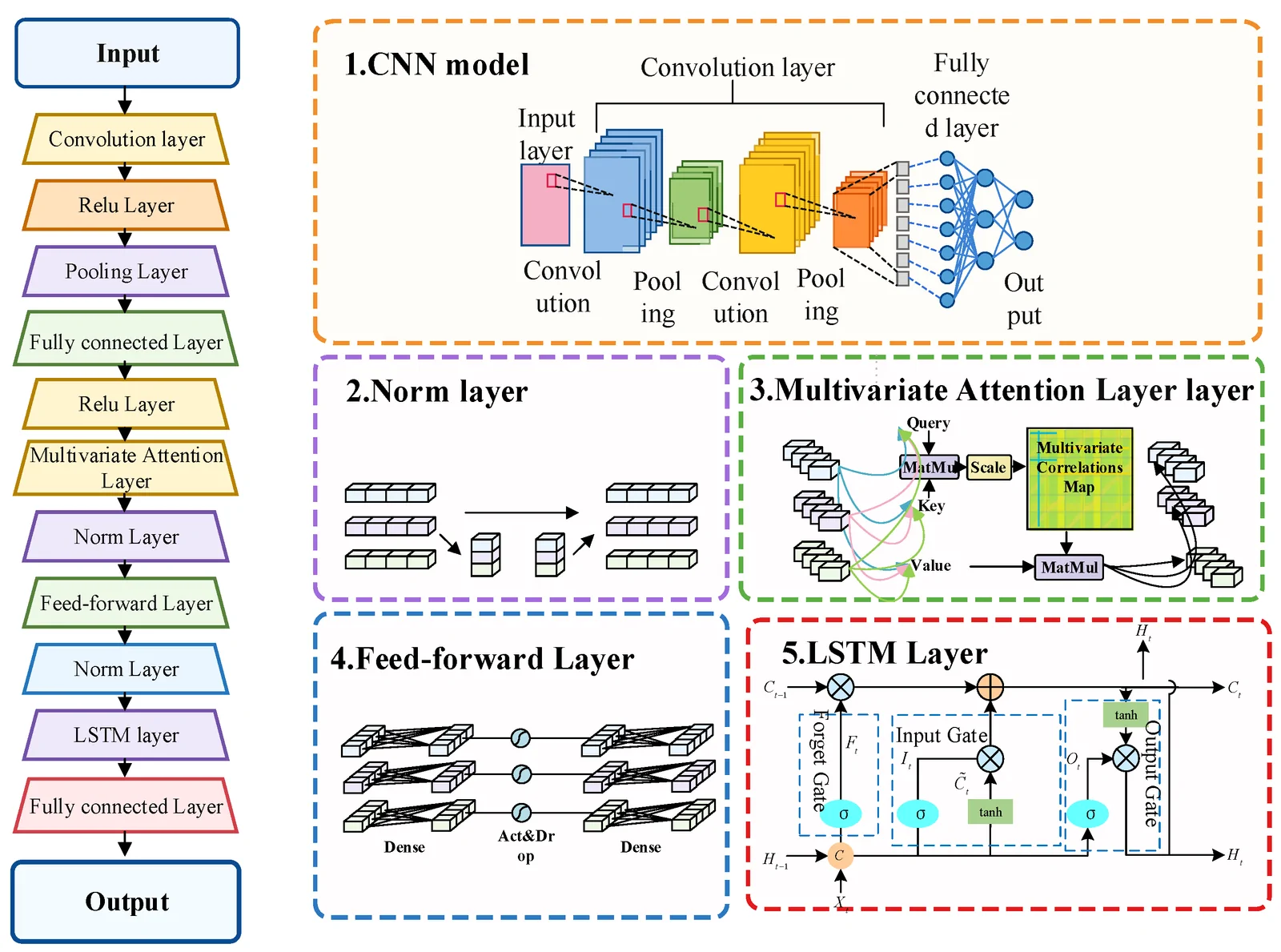
Framework of the CNN–iTransformer–LSTM model.

Figure 6 presents the overall Pearson–HOA–VMD–CNN–iTransformer–LSTM forecasting framework. The framework integrates the Pearson correlation coefficient (PCC), hiking optimization algorithm (HOA), VMD, CNN, iTransformer, and LSTM. It takes meteorological variables and historical wind power as inputs. Within the forecasting models, CNN extracts local fluctuation patterns. The iTransformer represents individual variable sequences as variate tokens and applies self-attention to capture global dependencies among meteorological factors and wind power. LSTM subsequently learns long-term temporal information. Finally, the extracted deep features are fed into a fully connected layer, and the corresponding predictions are generated using a regression layer. These predictions are subsequently inverse-standardized to restore them to the original power scale, yielding the final wind power forecasts.

Step 1: Data preprocessing. Missing values in the original dataset are identified and imputed using cubic spline interpolation. All meteorological variables and wind power observations are subsequently standardized using Z-score normalization.
Step 2: Feature selection and signal decomposition. PCC is calculated between each meteorological variable and wind power to remove weakly correlated or redundant inputs. Using the HOA–VMD, the original wind power sequence is decomposed into several modal components with different frequency characteristics.
Step 3: Use HOA to select hyperparameters of the CNN–iTransformer–LSTM network.
Step 4: Multilevel feature extraction. CNN extracts local fluctuation patterns, whereas iTransformer captures global variable dependencies using variate-wise self-attention. LSTM further captures long-term dependencies in the wind power data.
Step 5: Forecast generation and reconstruction. The extracted deep features are fed into a fully connected layer, and the corresponding predictions are generated through a regression layer. These predictions are subsequently inverse-standardized to yield the final wind power forecasts.
Step 6: Performance evaluation. The predicted wind power values are compared with the corresponding observations. MAE, MAPE, RMSE, and R^2^ are calculated to quantify forecasting performance and compare the proposed model with the benchmark models.

### 3.3. Optimization of VMD and CNN–iTransformer–LSTM Parameters Based on HOA

#### 3.3.1. Hiking Optimization Algorithm

In the optimization process, each hiker represents a candidate solution in the search space. For the parameter optimization problem considered in this study, the position of a hiker can represent a group of model parameters, such as the VMD parameters *K* and α, or the hyperparameters of the CNN–iTransformer–LSTM prediction model. The position matrix of the hiker population can be expressed as follows:

(25) $$\beta =\left[\begin{array}{cccc} \beta_{1,1} & \beta_{1,2} & \ldots & \beta_{1,j}\\ \beta_{2,1} & \beta_{2,2} & \ldots & \beta_{2,j}\\ \vdots & \vdots & \ddots & \vdots \\ \beta_{i,1} & \beta_{i,2} & \ldots & \beta_{i,j} \end{array}\right]$$

where $\beta_{i,j}$ represents the value of decision variable *j* for hiker *i*. In the original implementation of HOA, the hiker population is initialized as a matrix with the dimension of *I* × *j*.

At the beginning of the optimization process, the positions of hikers are randomly initialized within the upper and lower bounds of the decision variables. The initialization formula is given as follows:

(26) $$\beta_{i,t}=\phi_{j}^{1}+r_{j}\left(\phi_{j}^{2}-\phi_{j}^{1}\right)$$

where $\beta_{i,t}$ denotes the position of hiker *i* at iteration *t*, $r_{j}$ is a uniformly distributed random variable within the interval [0,1], and $\phi_{j}^{1}$ and $\phi_{j}^{2}$ represent the lower and upper bounds of decision variable *j*, respectively. This random initialization helps improve the diversity of candidate solutions in the early search stage.

The movement of hikers is determined using Tobler’s hiking function. This function calculates the walking velocity of a hiker according to the slope of the terrain. In HOA, the hiking velocity affects the step size of candidate solution updating and further influences the search behavior of the algorithm. The hiking velocity is defined as follows:

(27) $$W_{i,t}=6e^{-3.5\left|S_{i,t}+0.05\right|}$$

where $W_{i,t}$ represents the velocity of hiker i at iteration t and $S_{i,t}$ denotes the slope of the trail or terrain. The slope is calculated as follows:

(28) $$S_{i,t}=\frac{dh}{dx}=\tan\theta_{i,t}$$

where dh and dx denote the elevation difference and the horizontal distance covered by the hiker, respectively. $\theta_{i,t}$ represents the inclination angle of the terrain experienced by hiker i at iteration t, and its value lies within [0, 50°].

HOA uses both the social cooperation among hikers and the individual search ability of each hiker. During each iteration, the hiker with the best fitness value is regarded as the lead hiker. Other hikers update their velocities according to the position of the lead hiker, their own current positions, and the sweep factor. The updated velocity of hiker i is calculated as follows:

(29) $$W_{i,t}=W_{i,t-1}+\gamma_{i,t}\left(\beta_{best}-SF_{i,t}\beta_{i,t}\right)$$

where $W_{i,t}$ and $W_{i,t-1}$ denote the current velocity and the velocity at the previous iteration of hiker i, respectively; $y_{i,t}$ is a uniformly distributed random variable within [0, 1]; $\beta_{best}$ represents the position of the lead hiker; and $SF_{i,t}$ denotes the sweep factor, whose value lies within [1,3]. The sweep factor regulates how far other hikers move from the lead hiker, helping keep the search on promising regions.

After the velocity is updated, the position of each hiker is updated according to the following:

(30) $$\beta_{i,t+1}=\beta_{i,t}+W_{i,t}$$

where $\beta_{i,t+1}$ denotes the updated position of hiker i at iteration t+1. Using this updating rule, the candidate solutions are continuously adjusted, and the population gradually moves toward the optimal region of the search space.

In the HOA procedure, the fitness values of all hikers are evaluated at each iteration. The hiker with the best fitness value is selected as the lead hiker, and its position is used to guide the movement of the remaining hikers. The lead hiker is re-determined in every iteration to ensure that the current optimal solution continuously guides the population search. This iterative process continues until the maximum number of iterations is reached, and the best solution obtained during the search process is returned as the final optimization result.

#### 3.3.2. Parameter Optimization for Variational Modal Decomposition

VMD performance is primarily governed by the mode number K and penalty factor α. An unsuitable K may cause mode mixing or redundant components, while an inappropriate α can produce excessive smoothing or frequency overlap. Their joint selection is therefore essential for reliable decomposition.

**Figure 7:**
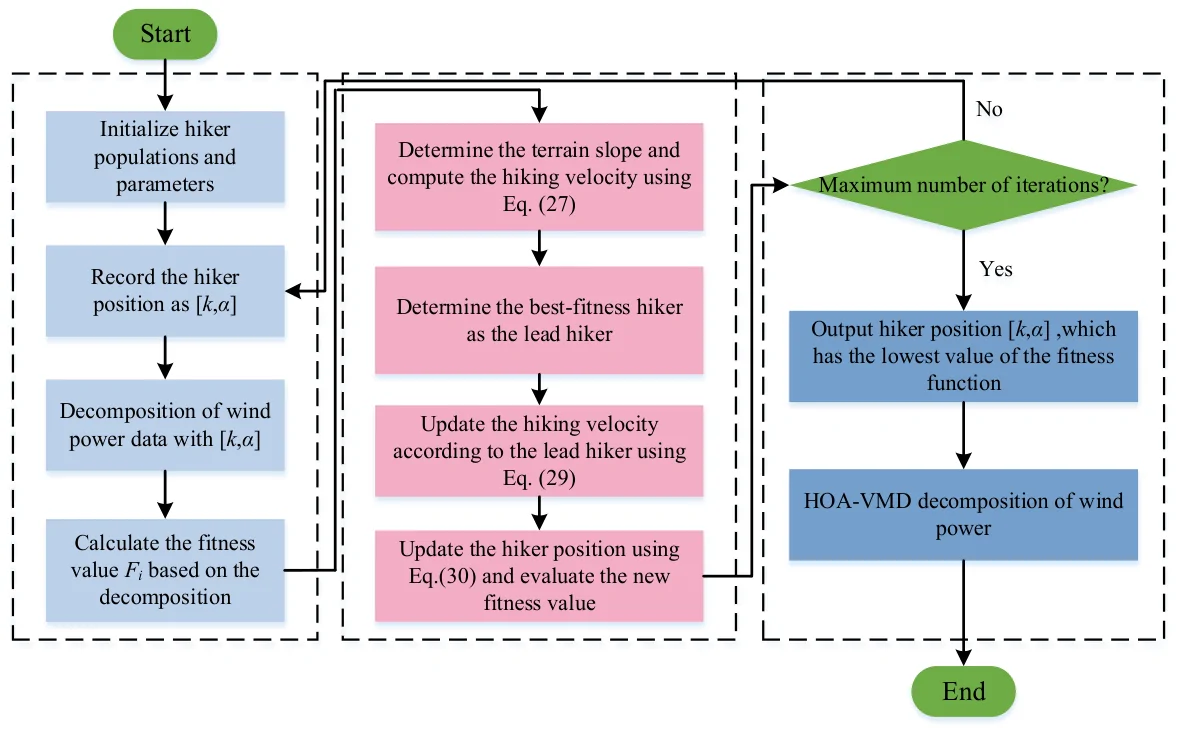
Flowchart of HOA-optimized VMD.

In this study, HOA selects K and α by minimizing a fitness function that combines envelope entropy with kurtosis. Envelope entropy quantifies disorder in a component; lower values indicate a more concentrated and regular signal. Kurtosis measures impulsive or sharp variations and is included to discourage excessive smoothing. Figure 7 shows the HOA–VMD search procedure.

After VMD decomposition, the envelope signal $a_{i}\left(j\right)$ of modal component $IMF_{i}\left(j\right)$ is obtained using Hilbert demodulation. Its normalized form can be expressed as follows:

(31) $$P_{ij}=\frac{a_{i}\left(j\right)}{\sum_{j=1}^{n}a_{i}\left(j\right)}$$

where $a_{i}\left(j\right)$ is the envelope of VMD mode $IMF_{i}\left(j\right)$ obtained using Hilbert demodulation and $P_{ij}$ is its normalized value. Indices i and j identify the mode and sampling point, respectively; n is the mode length.

The envelope entropy $E_{i}$ of modal component i can be expressed as follows:

(32) $$E_{i}=-\sum_{j=1}^{n}P_{ij}\ln P_{ij}$$

where $E_{i}$ denotes the envelope entropy of modal component i. A smaller envelope entropy indicates a more concentrated energy distribution, lower signal complexity, and more stable decomposition performance.

The kurtosis $\kappa_{i}$ of modal component i can be expressed as follows:

(33) $$\kappa_{i}=\frac{1}{n}\sum_{j=1}^{n}{\left(\frac{IMF_{i}\left(j\right)-\mu_{i}}{\sigma_{i}}\right)}^{4}$$

where $\kappa_{i}$ denotes the kurtosis of modal component i; $IMF_{i}\left(j\right)$ represents the value of modal component i at sampling point j; and $\mu_{i}$ and $\sigma_{i}$ represent the mean and standard deviation of modal component $IMF_{i}\left(j\right)$, respectively. A larger kurtosis value indicates more prominent peak or abrupt change characteristics in the signal. The introduction of kurtosis can prevent excessive smoothing during VMD decomposition and preserve key fluctuation information in the wind power sequence.

Accordingly, HOA minimizes the following combined fitness criterion, which favors lower envelope entropy and higher kurtosis:

(34) $$F_{i}=E_{i}+\frac{1}{\left|\kappa_{i}\right|}$$

where $F_{i}$ denotes the fitness value of modal component i, $E_{i}$ represents the envelope entropy, and $\kappa_{i}$ represents the kurtosis.

For each candidate parameter pair (*K*, *α*), the envelope entropy and kurtosis are calculated for each IMF. The minimum value of the combined mode-level criterion is used as the overall fitness, as defined below.

(35) $$F\left(K,\alpha \right)=\underset{i=1,...,K}{\min}F_{i}$$

HOA searches for the parameter pair that minimizes $F\left(K,\alpha \right)$.

#### 3.3.3. Hyperparameter Optimization of CNN–iTransformer–LSTM

The forecasting performance of CNN–iTransformer–LSTM depends on the joint configuration of its network parameters. HOA therefore searches six influential hyperparameters: the position-encoding dimension, number of attention heads, number of attention keys, number of LSTM neurons, dropout rate, and learning rate. MAE defines the fitness score.

**Figure 8:**
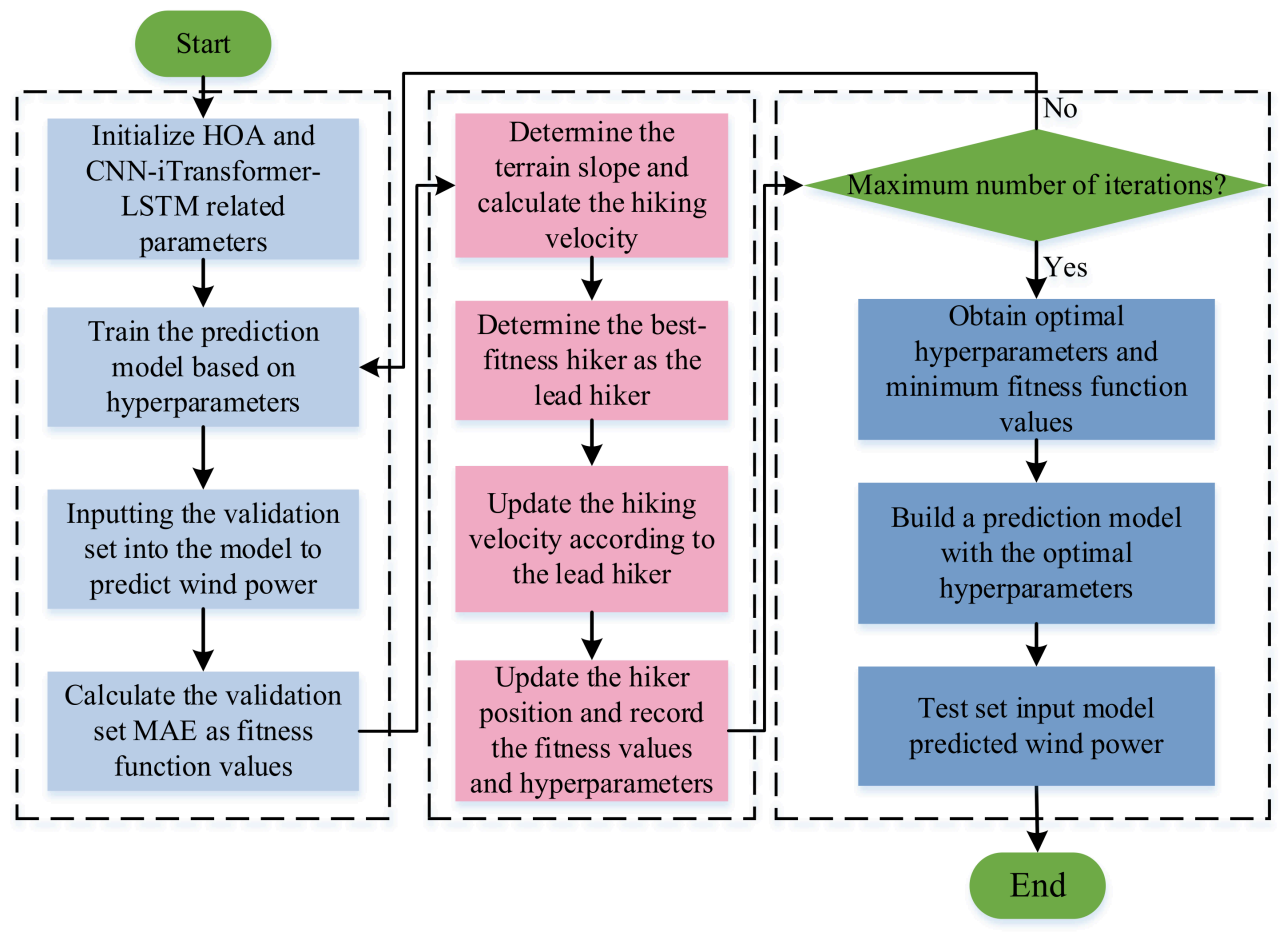
Flowchart of HOA-optimized CNN–iTransformer–LSTM.

Manual hyperparameter selection requires extensive trial and may overlook effective parameter interactions. Therefore, the HOA algorithm is adopted to search for the optimal hyperparameter combination automatically. HOA was applied separately to optimize the VMD parameters and the forecasting-network hyperparameters. After decomposition, the network hyperparameters were optimized independently. Each IMF was then predicted by its individually optimized CNN–iTransformer–LSTM network, and the component predictions were summed to reconstruct the wind power forecast. Figure 8 illustrates this optimization procedure.

## 4. Data Input and Evaluation Metrics

### 4.1. Input Processing of Data

#### 4.1.1. Data Standardization

Meteorological variables and wind power differ substantially in their units and numerical ranges. Each input variable is therefore standardized using the Z-score method before model training. The standardized value is calculated as follows:

(36) $$X_{nor}=\frac{X-\mu }{\sigma }$$

where X and $X_{nor}$ denote the original and standardized values, respectively. The mean μ and standard deviation σ are calculated separately for each variable using training data only.

#### 4.1.2. Pearson Correlation Coefficient

Using every recorded meteorological variable may introduce redundant inputs. The Pearson correlation coefficient (PCC) is therefore used to quantify the linear association between each continuous meteorological variable and wind power output. Variables exhibiting stronger correlations are retained, whereas weakly related features are excluded.

Assume that the wind power dataset contains m sampling moments and n feature variables. The data matrix can be expressed as follows:

(37) $$X={\left(x_{ij}\right)}_{m\times n}$$

where $x_{ij}$ represents the recorded value associated with sample i and variable j. In the wind power forecasting dataset, $x_{a}$ denotes the meteorological variable contained in column a, such as wind speed, wind direction, air temperature, atmospheric pressure, or relative humidity, while $x_{p}$ denotes the wind power sequence contained in column p. To investigate the linear correlation between a selected meteorological variable $x_{a}$ and the wind power sequence $x_{p}$, the Pearson correlation coefficient is denoted as $\rho \left(a,p\right)$, and its calculation formula is expressed as follows:

(38) $$\rho \left(a,p\right)=\frac{\sum_{i=1}^{m}\left(x_{a,i}-{\bar{x}}_{a}\right)\left(x_{p,i}-{\bar{x}}_{p}\right)}{\sqrt{\sum_{i=1}^{m}{\left(x_{a,i}-{\bar{x}}_{a}\right)}^{2}\sum_{i=1}^{m}{\left(x_{p,i}-{\bar{x}}_{p}\right)}^{2}}}$$

where m denotes the number of samples in the wind power dataset. $x_{a,i}$ represents the value of the selected meteorological variable $x_{a}$ in sample i, and $x_{p,i}$ denotes the corresponding wind power value in the same sample. ${\bar{x}}_{a}$ and ${\bar{x}}_{p}$ represent the mean values of the meteorological variable $x_{a}$ and the wind power sequence $x_{p}$, respectively.

PCC lies between −1 and 1. Values near 1 or −1 indicate strong positive or negative linear association, respectively, whereas values near zero indicate little linear association. Feature relevance is assessed by the magnitude of the coefficient.

### 4.2. Evaluation Indicator

Forecasting performance is quantified using four metrics: the coefficient of determination (R^2^), root mean square error (RMSE), mean absolute error (MAE), and mean absolute percentage error (MAPE). Their formulas are as follows:

(39) $$R^{2}=1-\frac{\sum_{i=1}^{n}{\left(y_{i}-{\hat{y}}_{i}\right)}^{2}}{\sum_{i=1}^{n}{\left(y_{i}-{\bar{y}}_{i}\right)}^{2}}$$

(40) $$\mathrm{RMSE}=\sqrt{\frac{1}{n}\sum_{i=1}^{n}{\left(y_{i}-{\hat{y}}_{i}\right)}^{2}}$$

(41) $$\mathrm{MAE}=\frac{1}{n}\sum_{i=1}^{n}\left|y_{i}-{\hat{y}}_{i}\right|$$

(42) $$\mathrm{MAPE}=\frac{1}{n_{nz}}\sum_{i=1}^{n}\left|\frac{y_{i}-{\hat{y}}_{i}}{y_{i}}\right|$$

where n is the number of test samples, $n_{nz}$ is the number of samples with nonzero observed power, $y_{i}$ is the true wind power value, ${\hat{y}}_{i}$ is the predicted wind power value, and $\bar{y}$ is the mean value of all true wind power values. Samples with zero observed power are excluded only when calculating MAPE. A value of R^2^ closer to 1 indicates that the model has a stronger ability to fit the variation trend of wind power. Lower RMSE, MAE, and MAPE indicate smaller prediction errors and higher prediction accuracy.

Model predictions are restored to the original wind power scale before the evaluation metrics are computed.

## 5. Simulation and Analysis

### 5.1. Parameter Setting and Environment Configuration

VMD was implemented using MATLAB R2024b. The Lagrange multiplier update rate τ was 0.01. The absolute and relative convergence tolerances were $5\times 10^{-6}$ and $5\times 10^{-3}$, respectively. The maximum iteration count was 100. Central frequencies were initialized using the peaks method. When insufficient spectral peaks were available, the remaining central frequencies were initialized randomly. The initial IMFs and Lagrange multiplier were zero.

The CNN contains a single one-dimensional convolutional layer with a kernel size of 1 and 32 filters. The attention module contains two self-attention layers with residual connections and layer normalization, with an input and output channel dimension of 20. The additional learnable positional embedding layer also has an output dimension of 20. The LSTM consists of one layer. Training uses Adam and a squared-error regression loss, with a batch size of 50 and a maximum of 70 epochs. Validation-based early stopping is not enabled. The proposed network is optimized separately for each IMF using HOA, whereas the benchmark models are manually tuned.

The HOA algorithm utilizes 10 agents, with a maximum iteration limit of 30. For the VMD-based baseline models, the mode number K and penalty factor α were fixed at five and 1500, respectively. The basic parameters of HOA–VMD are as follows: the search range for k is [1, 12], and the search range for α is [50, 2500]. The basic parameters of HOA–CNN–iTransformer–LSTM are as follows: the search range for positional encoding dimensions is [1, 128], search range for number of attention heads is [1, 128], search range for the number of attention keys is [1, 128], and search range for LSTM neuron count is [1, 512]. The search range for learning rate is [0.001, 0.01], and the search range for drop rate is [0.001, 0.5]. The time step for input data is consistent across all prediction models and is set to five. The batch size is 50 for the input model. The optimizer used is Adam. The experiments were implemented in MATLAB R2024b on Windows 11. Computation used an Intel Core i9-14900HX processor, an NVIDIA GeForce RTX 5070 graphics card, and 16 GB of RAM.

### 5.2. Data Description and Correlation Analysis

#### 5.2.1. Data Description

The evaluation used operational data from two wind farms in different regions of China with installed capacities of 99 and 200 MW. Records were collected every 15 min throughout 2020 and included generated power, wind speed and direction at multiple heights, air temperature, atmospheric pressure, and relative humidity. The 99 MW wind farm comprises 50 GW1500/85 turbines and 12 H93 L-2.0mw turbines, with hub heights of 85.0 m. The 200 MW wind farm comprises 67 GW3000/110 turbines with a hub height of 120.0 m.

**Figure 9:**
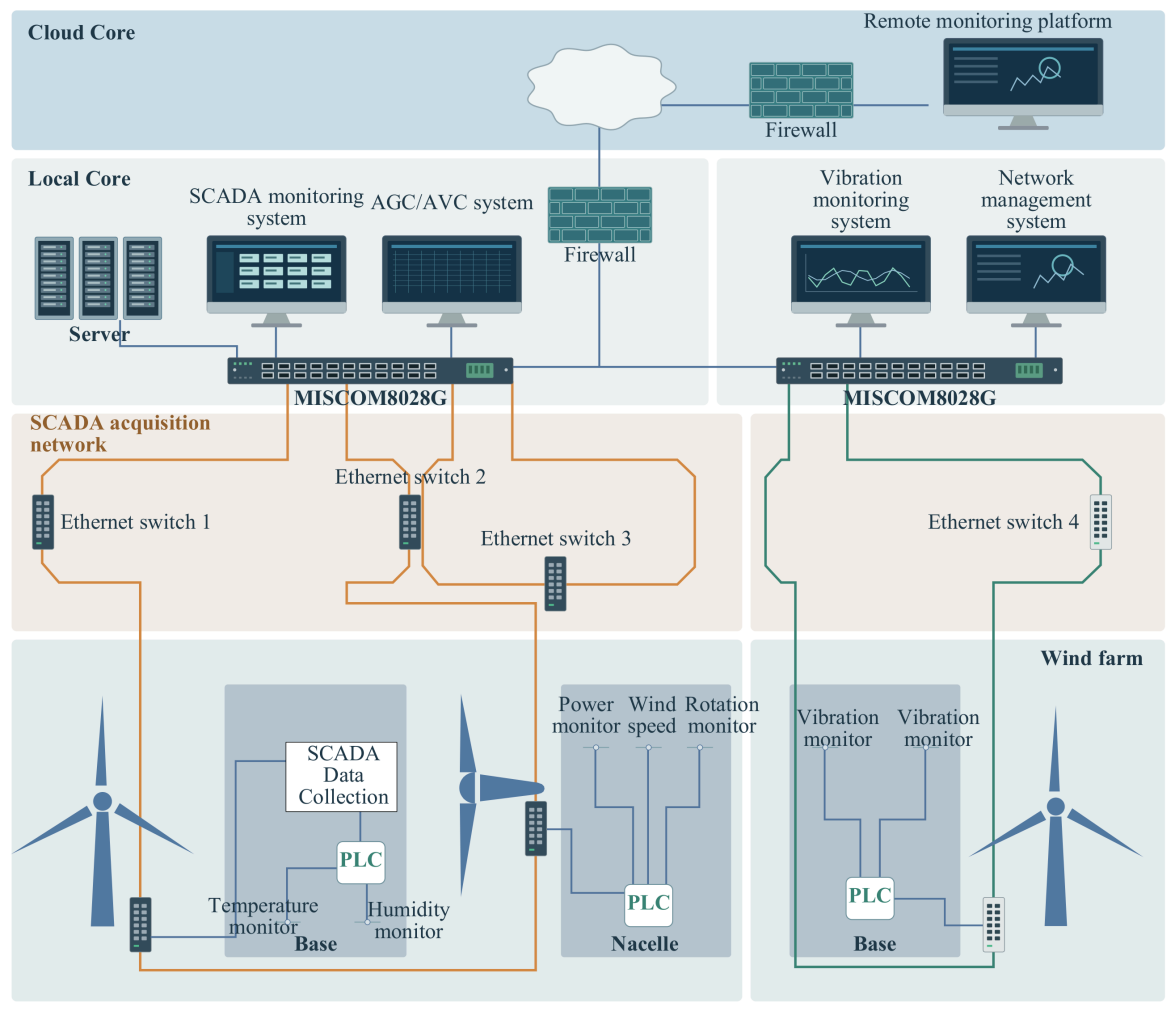
Sensor architecture and data collection process of the wind farms.

Four seasonal subsets were constructed. March and November records from the 99 MW site formed Datasets 1 and 2, while May and August records from the 200 MW site formed Datasets 3 and 4. Each subset was partitioned chronologically into training, validation, and test portions in an 8:1:1 ratio, before preprocessing and VMD decomposition. Table 1 summarizes the descriptive statistics of wind power output for the four datasets, including the maximum, minimum, mean, standard deviation, estimated capacity factor, and proportion of zero-power samples. Figure 9 provides sensor architecture and data collection process of the wind farms.

**Figure 10:**
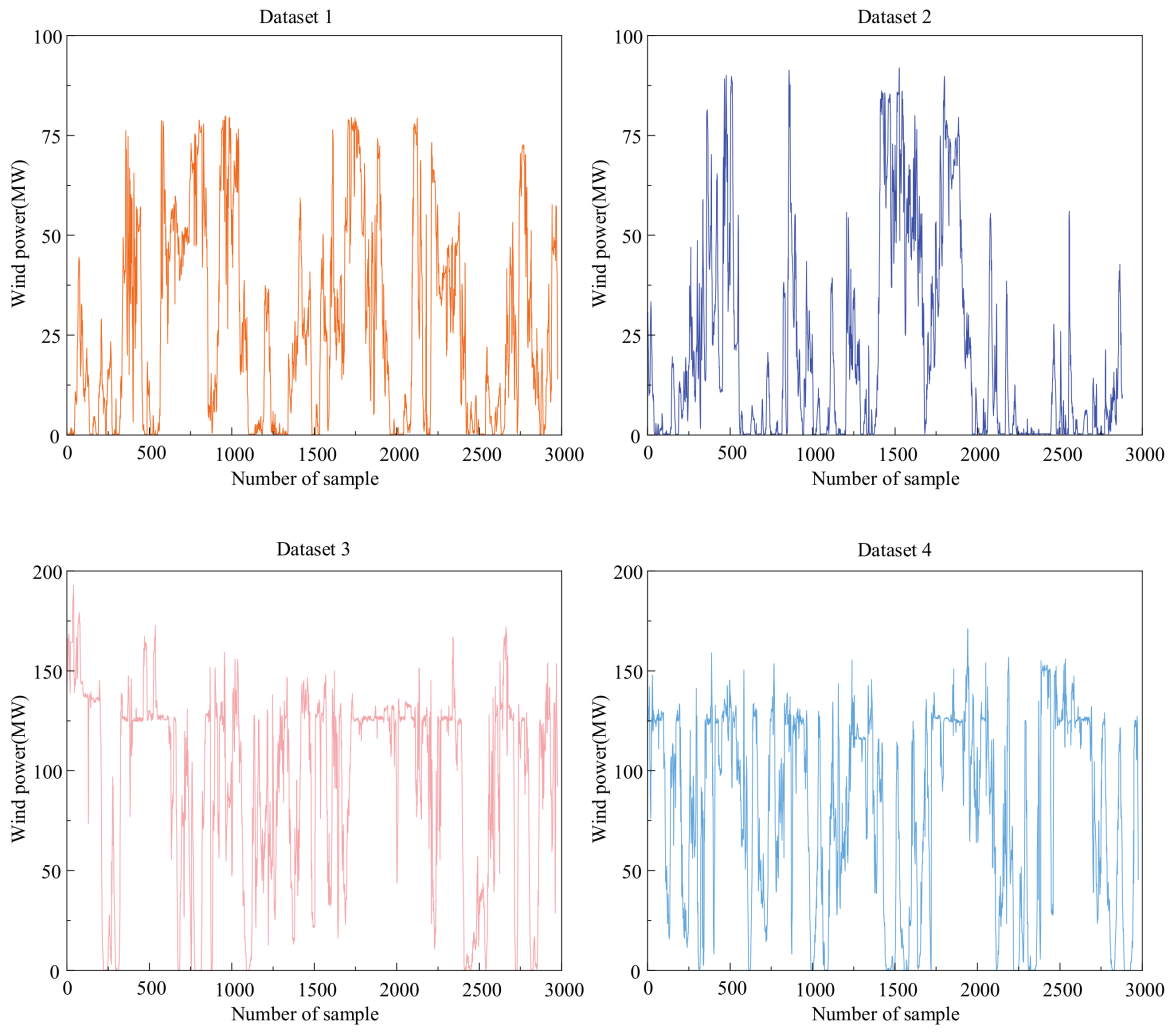
Wind power data.

**Figure 11:**
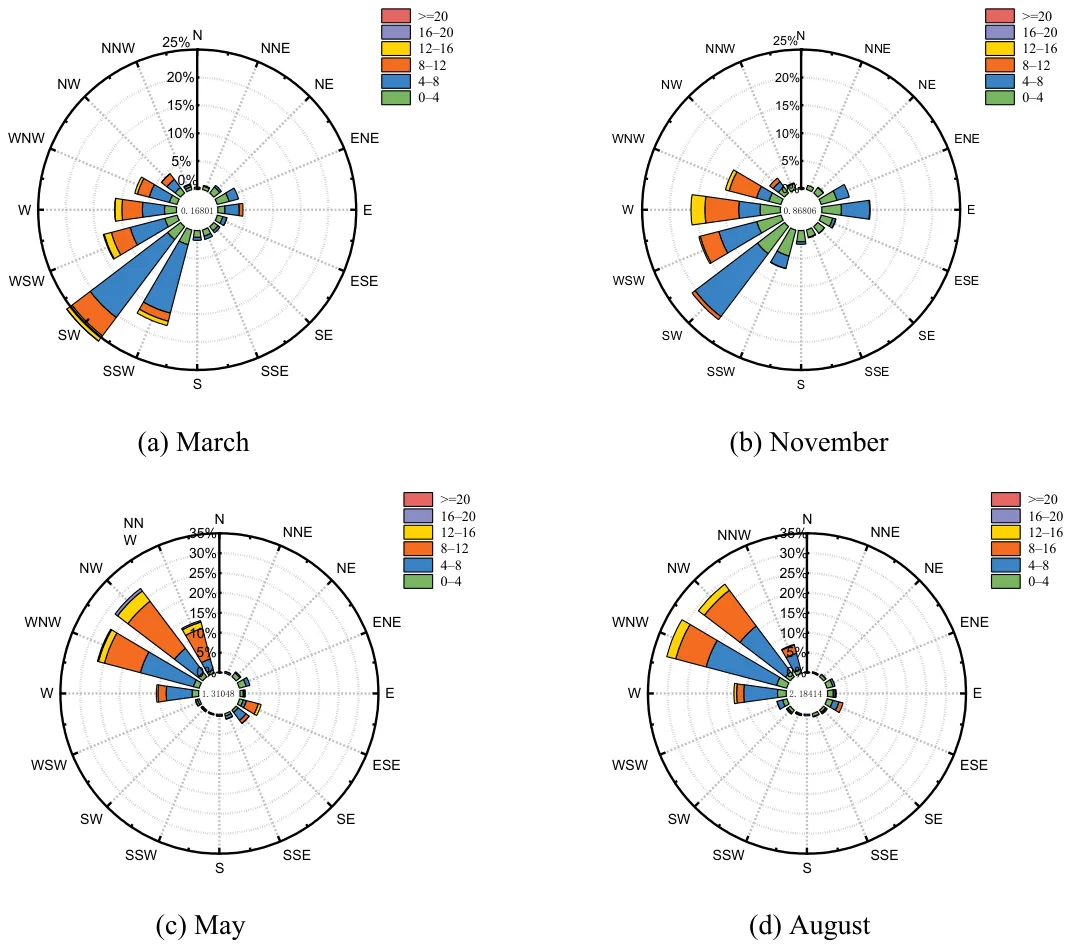
Wind rose diagram.

Figure 10 displays the four power series and illustrates their markedly different fluctuation patterns. Wind rose plots in Figure 11 summarize the joint distributions of direction and speed.

The wind roses describe how frequently each speed interval occurs within 16 directional sectors. Sector length represents frequency and color identifies the speed range.

Figure 11 shows that March winds originate predominantly from the southwest and south-southwest, with the southwest sector occurring most frequently. Most wind speeds lie between 4 and 12 m/s, while observations above 12 m/s are uncommon. November exhibits a similar pattern, with southwest, west-southwest, and west as the prevailing directions. Wind speeds are again concentrated between 4 and 12 m/s, although some observations reach 12–16 m/s.

A different directional regime is observed in May, when northwest and west-northwest winds become dominant. Speeds mainly range from 4 to 16 m/s, with only a few observations exceeding 16 m/s. August shows a comparable northwesterly pattern, and the northwest sector has the highest frequency. The 8–12 m/s interval accounts for a particularly large proportion of the August observations. Overall, March and November are characterized by southwesterly winds, whereas northwesterly winds prevail in May and August.

#### 5.2.2. Correlation Analysis

**Figure 12:**
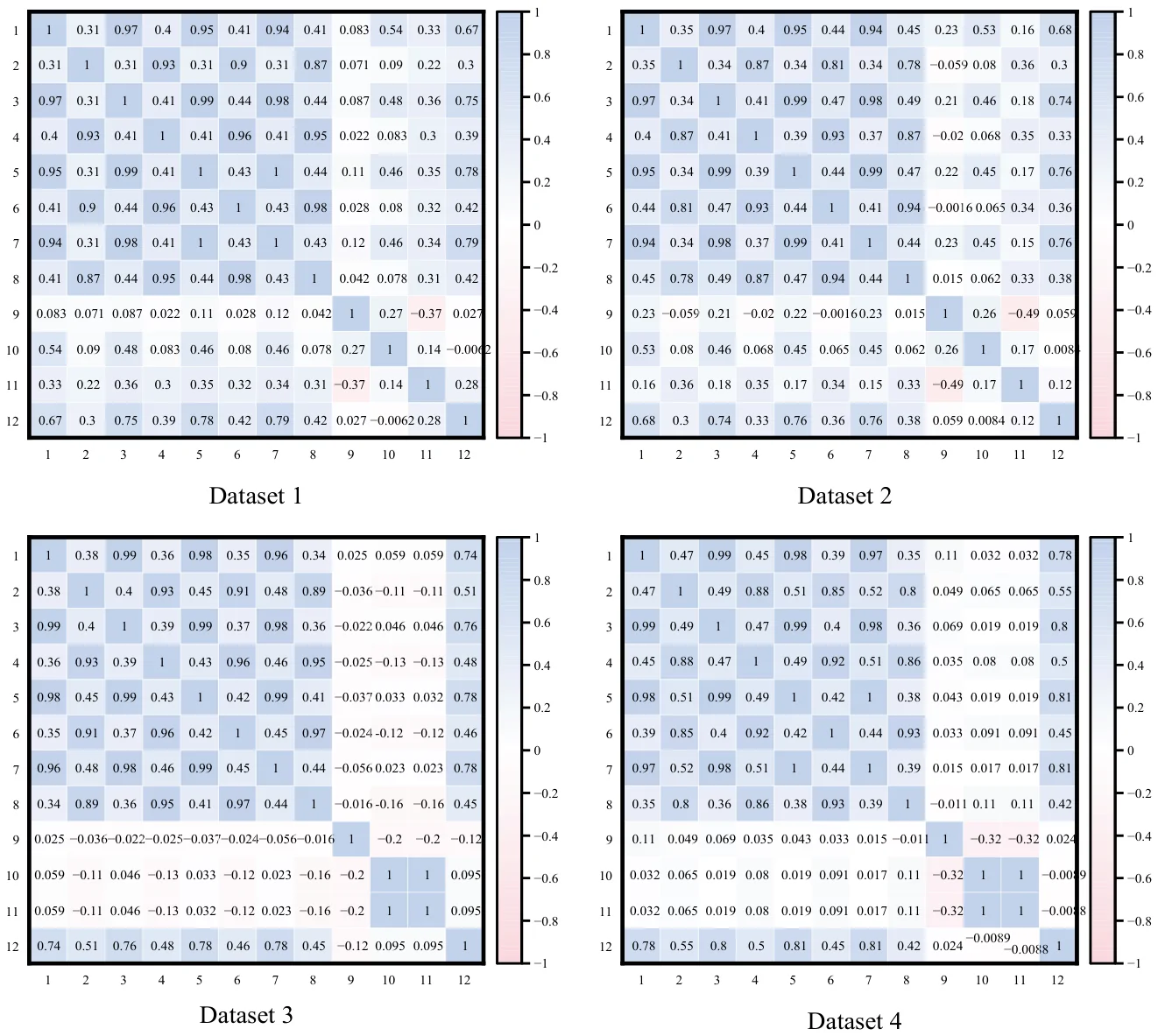
Pearson correlation analysis.

For each candidate meteorological variable, the Pearson correlation coefficient (PCC) is calculated against wind power output to assess their linear relationship. Features with relatively strong correlations are retained, while weakly related variables are excluded. This screening procedure reduces input redundancy and computational burden. The results are shown in Figure 12.

The heatmap analysis indicates that wind speeds at different heights exhibit strong positive correlations with wind power. In particular, the correlation coefficients between wind power and wind speed at 10 m, 30 m, 50 m, and hub height remain at relatively high levels, suggesting that wind speed is the dominant factor affecting variations in wind power output. Wind directions at different heights also show a certain degree of correlation with wind power; however, their correlation levels are generally lower than those of wind speed. In addition, meteorological factors such as air temperature, atmospheric pressure, and relative humidity show relatively weak correlations with wind power, and their effects vary among different datasets. Overall, the correlation analysis results demonstrate that wind speed and wind direction have significant impacts on wind power generation, with wind speed playing a more prominent role, while the influence of other meteorological factors is comparatively limited. Table 2 and Table 3 report the Pearson correlation coefficients between meteorological variables and wind power for the four datasets.

Indices 1–12 in the correlation map denote, in order, wind speed and direction at 10 m, 30 m, 50 m, and hub height, followed by air temperature, atmospheric pressure, relative humidity, and wind power.

Across the four datasets, wind speeds at different heights exhibit the strongest positive linear correlations with wind power. Wind direction variables show weaker associations, while air temperature, atmospheric pressure, and relative humidity generally have lower absolute correlation coefficients. Accordingly, meteorological variables are ranked by the absolute value of their Pearson correlation coefficients with wind power, and the five highest-ranked variables are retained for each dataset.

### 5.3. Experimental Results and Analysis

#### 5.3.1. Comparison of Benchmark Models

In this study, HOA is used to select the VMD parameters for each dataset. The resulting decomposition separates the wind power series into frequency-specific components for subsequent forecasting.

Table 4 lists the HOA-selected VMD parameters for each dataset. Table 5 reports the corresponding hyperparameter settings for the CNN–iTransformer–LSTM forecasting network.

**Figure 13:**
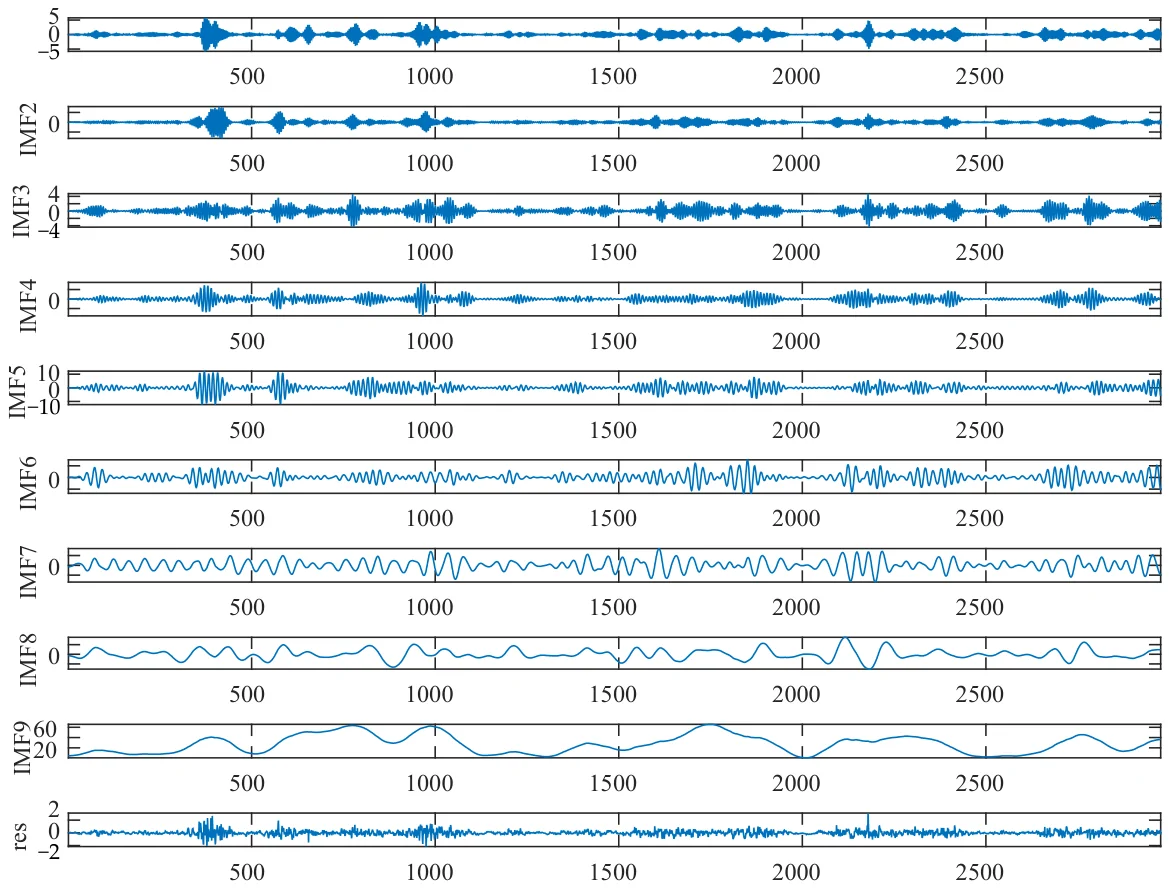
Decomposition results of wind power for HOA–VMD decomposition Dataset 1.

**Figure 14:**
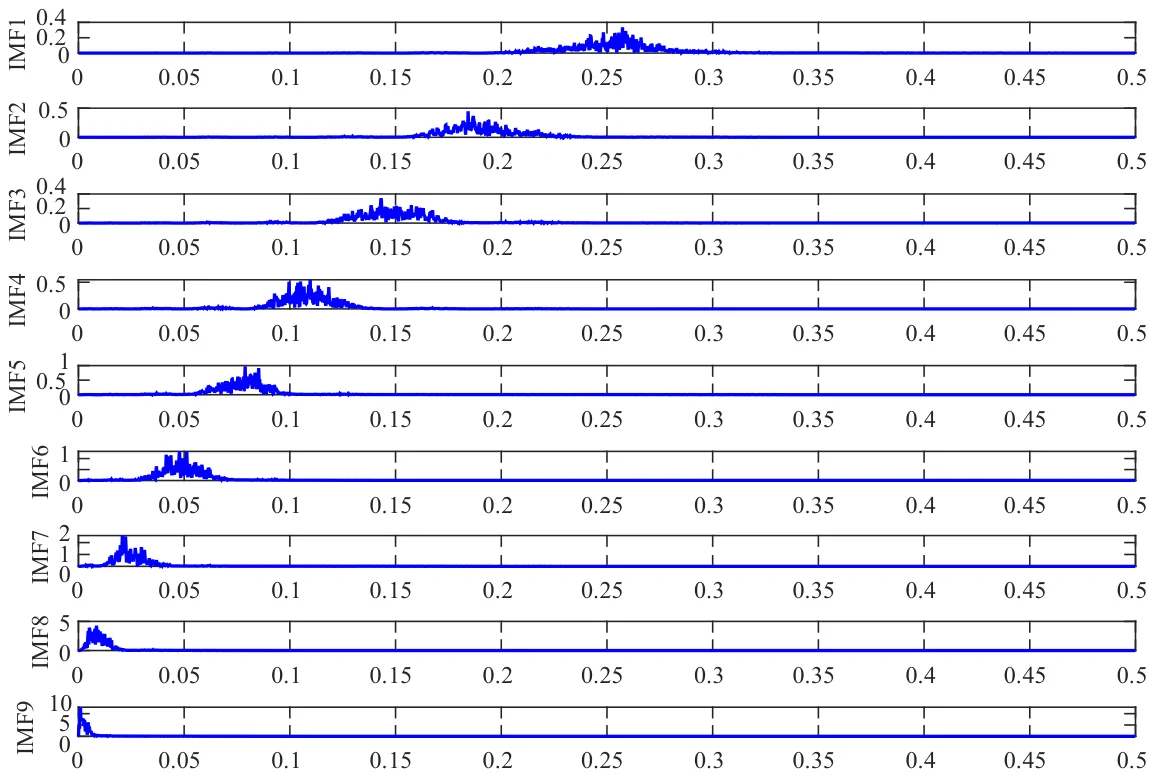
Spectrogram of the decomposition result.

The final decomposition results are shown in Figure 13. As illustrated by the spectrograms of the decomposed modal components in Figure 14, the obtained modes are arranged in descending order of dominant frequency. The center frequencies of different modal components are clearly separated, suggesting that HOA–VMD can effectively reduce modal mixing and improve the decomposition quality of wind power series.

To further verify the forecasting performance of the proposed model, several benchmark models were selected for comparative analysis. These models include single models, hybrid models, decomposed hybrid models, and HOA-optimized decomposed hybrid models. Specifically, CNN, LSTM, BiLSTM, and iTransformer were used as single models. CNN–LSTM, iTransformer–LSTM, and CNN–iTransformer–LSTM were adopted as hybrid models. VMD–CNN, VMD–LSTM, and VMD–CNN–iTransformer–LSTM were used as decomposed hybrid models. HOA–VMD–CNN–iTransformer–LSTM optimizes the VMD mode number K and penalty factor α, while retaining manually configured network hyperparameters. VMD–HOA–CNN–iTransformer–LSTM fixes K and α and optimizes the network hyperparameters. The proposed framework uses separate HOA searches to optimize the VMD parameters and network hyperparameters. The same PCC-selected meteorological inputs are used in these three configurations. Table 6 and Table 7 report MAE, MAPE, RMSE, and R^2^ for the evaluated models across Datasets 1–4.

**Figure 15:**
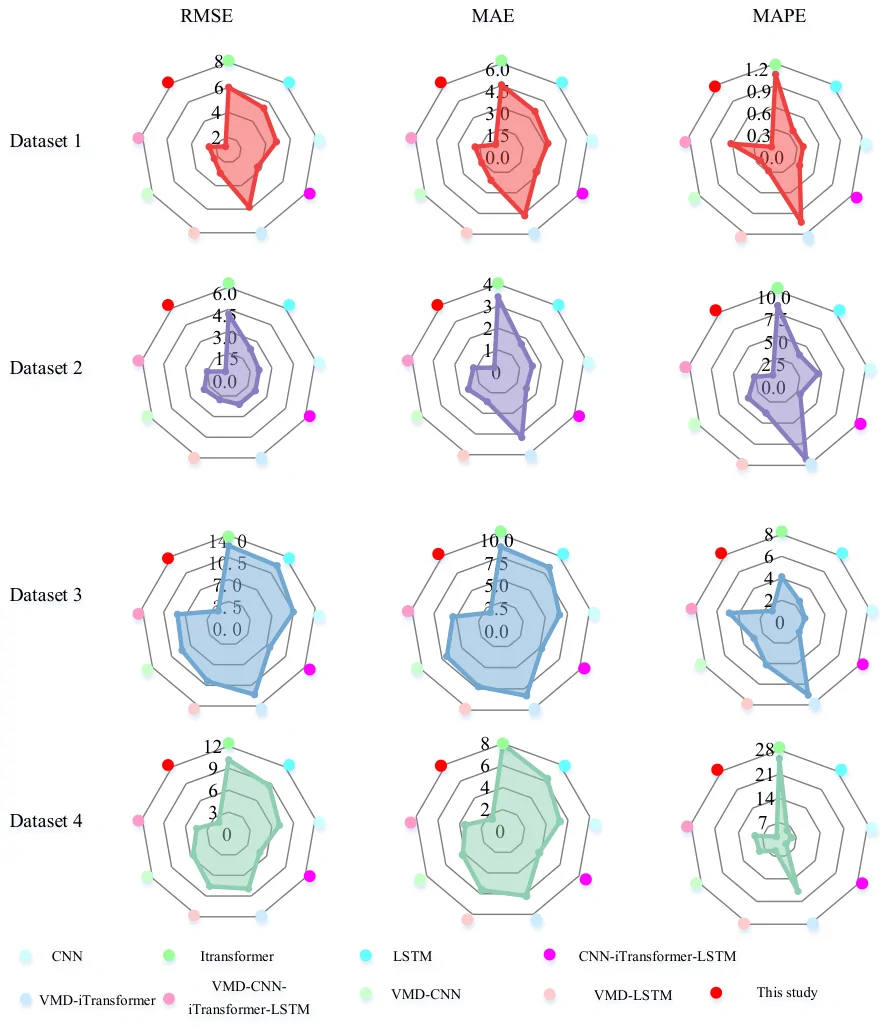
Evaluation metrics of the benchmark model prediction.

**Figure 16:**
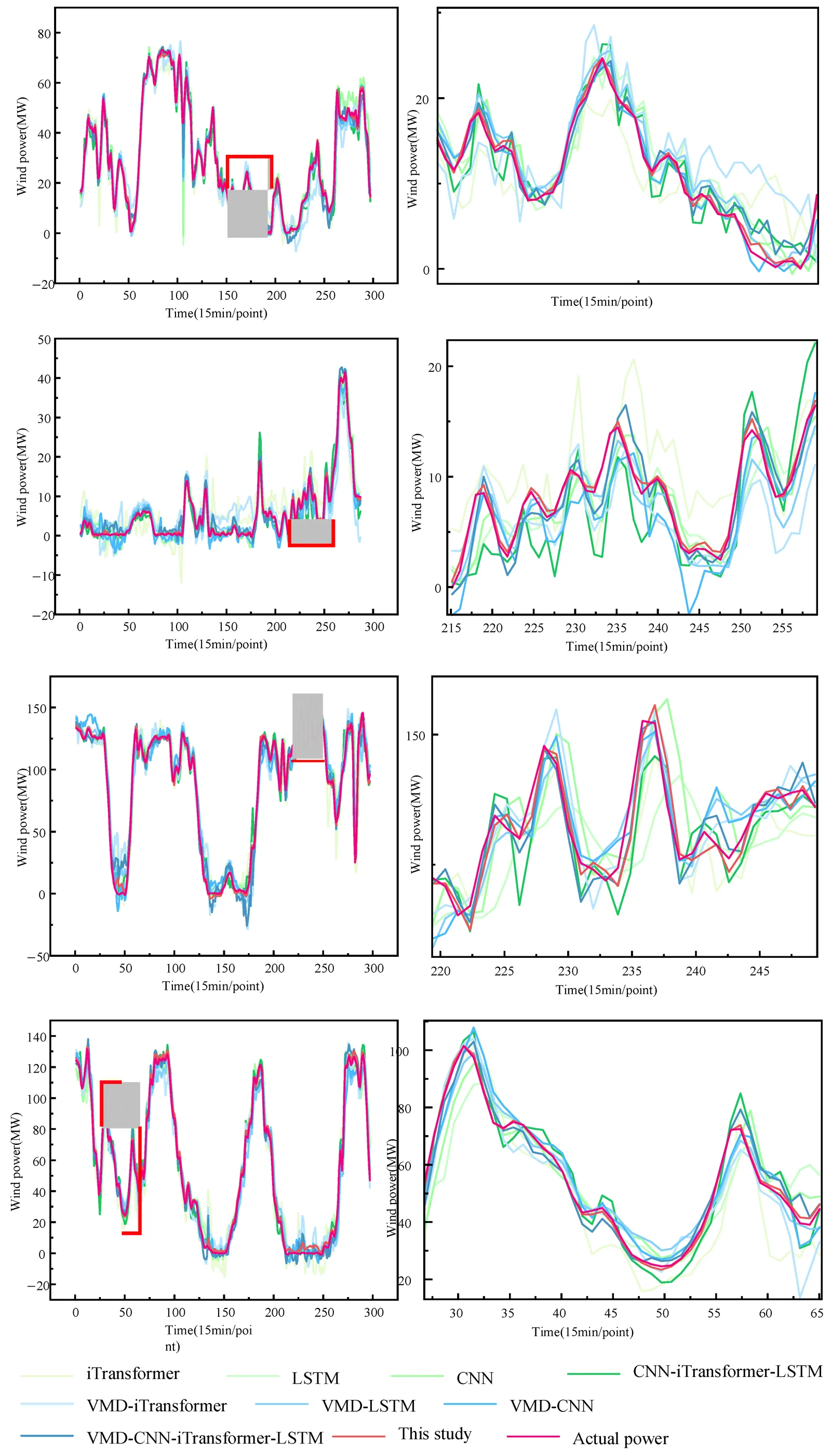
Comparison of benchmark model prediction result curves.

Figure 15 compares the evaluation metrics of all models across Datasets 1–4, while Figure 16 presents their prediction curves. The performance differences among different models under various datasets are further analyzed as follows:

(1) Dataset 1. The CNN–iTransformer–LSTM model produced lower RMSE and MAE values than the three individual networks. Its RMSE was 3.6627, representing reductions of 39.11%, 31.69%, and 24.71% relative to iTransformer, LSTM, and CNN, respectively.

The corresponding MAE reductions were 40.11%, 23.53%, and 15.47%, while R^2^ reached 0.9695. These results indicate that the fusion model can comprehensively extract local features, temporal dependencies, and global information from wind power sequences, thereby improving prediction accuracy.

After introducing VMD, the prediction performance of the LSTM and CNN models is further improved. Compared with the LSTM model, the RMSE, MAE, and MAPE of the VMD–LSTM model decrease by 45.10%, 41.49%, and 24.22%, respectively. VMD–CNN yielded corresponding reductions of 52.04%, 52.02%, and 37.01% relative to CNN, while R^2^ rose from 0.9462 to 0.9876. This improvement can be attributed to the ability of VMD to effectively decompose non-stationary wind power sequences, reduce noise interference, and alleviate the interaction among different frequency components, making the decomposed data more suitable for feature extraction by deep learning models. In addition, compared with the CNN–iTransformer–LSTM model without VMD, the RMSE and MAE of the VMD–CNN–iTransformer–LSTM model decrease by 29.32% and 32.74%, respectively.

The proposed model achieves the best prediction performance on Dataset 1, with MAE, MAPE, and RMSE values of 0.6255, 0.0848, and 1.3779, respectively, and an R^2^ value of 0.9957. Compared with the best-performing benchmark model without HOA optimization, VMD–CNN, the MAE, MAPE, and RMSE of the proposed model decrease by 59.65%, 64.90%, and 40.95%, respectively, while the R^2^ value increases from 0.9876 to 0.9957. Furthermore, compared with HOA–VMD–CNN–iTransformer–LSTM and VMD–HOA–CNN–iTransformer–LSTM, the proposed model reduces RMSE by 41.79% and 1.34%, respectively. Relative to the latter model, MAE and MAPE decrease by 38.25% and 43.77%, respectively.

CNN captures local power fluctuations, while iTransformer and LSTM further process the extracted features through attention and recurrent layers. VMD separates fluctuations at different frequencies, helping the hybrid network model the non-stationary power series.

(2) For Dataset 2, CNN has the lowest MAE and RMSE among the single models, whereas LSTM obtains the lowest MAPE; iTransformer alone is least accurate overall. The standalone iTransformer performed less consistently across datasets. On Dataset 2, its R^2^ was 0.7478, compared with 0.9178–0.9491 on the other three datasets. Relative to CNN, CNN–iTransformer–LSTM decreases MAE, MAPE, and RMSE by 6.09%, 36.68%, and 0.07%, respectively.

After VMD decomposition is introduced, the prediction performance of different models is generally further enhanced. Among the VMD-based hybrid models, VMD–CNN–iTransformer–LSTM achieves the best performance, with MAE, MAPE, and RMSE values of 1.1347, 2.6234, and 1.4828, respectively, and an R^2^ value of 0.9681. Compared with the CNN–iTransformer–LSTM model without VMD decomposition, its MAE, MAPE, and RMSE decrease by 23.95%, 9.63%, and 29.79%, respectively.

The proposed model obtains the optimal prediction results on Dataset 2, with MAE, MAPE, and RMSE values of 0.2650, 0.8372, and 0.3358, respectively, and an R^2^ value of 0.9984. Compared with the best-performing benchmark model without HOA optimization, VMD–CNN–iTransformer–LSTM, the MAE, MAPE, and RMSE of the proposed model decrease by 76.65%, 68.09%, and 77.35%, respectively, while the R^2^ value increases from 0.9681 to 0.9984. Compared with HOA–VMD–CNN–iTransformer–LSTM and VMD–HOA–CNN–iTransformer–LSTM, the proposed model achieves RMSE reductions of 75.78% and 54.94%, respectively. Relative to the latter model, MAE decreases by 49.44%, whereas MAPE increases from 0.7931 to 0.8372, corresponding to a relative increase of 5.56%.

The weaker performance of the standalone iTransformer on Dataset 2, despite better results on the other datasets, suggests that its effectiveness is dataset dependent. Combining it with CNN and LSTM improves feature representation, while VMD reduces the complexity of the mixed frequency input.

(3) For Dataset 3, CNN performs best among the individual models, recording MAE, MAPE, RMSE, and R^2^ values of 6.6002, 2.1360, 10.4734, and 0.9548, respectively. CNN–iTransformer–LSTM reduces MAE, MAPE, and RMSE by 21.09%, 14.22%, and 27.63%, respectively, while increasing R^2^ to 0.9763.

Among the VMD-based models without HOA optimization, VMD–CNN–iTransformer–LSTM achieves the lowest MAE and RMSE, with values of 5.3666 and 8.2814, respectively, and the highest R^2^ of 0.9718. However, VMD–CNN records the lowest MAPE of 2.9244.

Compared with the benchmark models, the proposed framework produced the most accurate forecasts, with an MAE of 1.8223, a MAPE of 1.3529, an RMSE of 2.5964, and an R^2^ of 0.9972. Compared with VMD–CNN–iTransformer–LSTM, it reduces MAE, MAPE, and RMSE by 66.04%, 72.09%, and 68.65%, respectively. Relative to CNN–iTransformer–LSTM, the corresponding reductions are 65.01%, 26.16%, and 65.75%, while R^2^ improves by 0.0209. Further comparisons with HOA–VMD–CNN–iTransformer–LSTM and VMD–HOA–CNN–iTransformer–LSTM show RMSE reductions of 52.35% and 31.01%, respectively. Compared with the latter model, the proposed framework also reduces MAE and MAPE by 24.81% and 7.33%, respectively.

For CNN–iTransformer–LSTM, both MAE and RMSE increase after introducing fixed-parameter VMD, indicating that decomposition is not uniformly beneficial under the selected configuration. MAPE additionally amplify errors at low observed power.

Power variations at the 200 MW farm are more complex. Notably, VMD alone did not improve every metric of the unoptimized hybrid model on this dataset, showing that decomposition must be matched with suitable network parameters.

(4) For Dataset 4, CNN is the best single-network baseline, while iTransformer alone is the least accurate. The CNN–iTransformer–LSTM RMSE of 4.9286 is 30.25% below that of CNN.

VMD–CNN–iTransformer–LSTM is the strongest decomposition-based benchmark without HOA optimization for MAE, RMSE, and R^2^, with values of 3.4844, 4.4331, and 0.9903, respectively. The proposed framework further reduces MAE, MAPE, and RMSE to 1.5137, 3.2337, and 2.0852, respectively, while increasing R^2^ to 0.9984. Its RMSE is 52.96% below VMD–CNN–iTransformer–LSTM and 57.69% below the undecomposed CNN–iTransformer–LSTM. Compared with HOA–VMD–CNN–iTransformer–LSTM and VMD–HOA–CNN–iTransformer–LSTM, the proposed model reduces RMSE by 47.90% and 6.57%, respectively. Relative to the latter model, MAE and MAPE decrease by 6.41% and 6.72%, respectively.

The improvement after adding VMD indicates that frequency separation also benefits the hybrid model under this dataset’s conditions. CNN, iTransformer, and LSTM then process the decomposed components jointly, improving the prediction of power.

Table 8 presents the training, validation, and test MAE of the proposed model. Dataset 3 shows the largest difference among the three subsets: its training MAE is 0.85850, while its validation and test MAE values are 2.48247 and 1.82226, respectively. The test MAE is also higher than the training MAE on Dataset 1, but not on Datasets 2 or 4. These results indicate that generalization performance varies across datasets.

#### 5.3.2. Multi-Step Prediction Experiments

Multi-step forecasts were generated using a multiple input multiple output strategy. A separate network was trained for each IMF and the residual component, with each network jointly predicting the next three time steps, corresponding to 15, 30, and 45 min ahead. The component forecasts were then summed to obtain the wind power forecasts. The residual in Step 6 denotes the difference between the original signal and the sum of the extracted IMFs, rather than a prediction error. Predicted power is not recursively fed back into the model. Only historical wind power and meteorological observations available at the forecast origin were supplied as inputs. No future observed meteorological variables were used.

To assess the value of the proposed forecasting framework beyond simply carrying forward the latest observation, a persistence baseline was included. Let t denote the forecast origin and $P_{t}$ the latest observed wind power. The baseline prediction is ${\hat{P}}_{t+h}=P_{t}$, where h = 1, 2, 3 corresponds to 15, 30, and 45 min ahead, respectively. This baseline requires neither model training nor future meteorological observations. Table 9 compares the persistence baseline with the proposed model across the four datasets at forecast horizons of 15, 30, and 45 min.

As shown in Table 9, the proposed model achieved lower MAE and RMSE and higher R^2^ than persistence across all four datasets and forecast horizons. The proposed model does not outperform persistence on every metric because MAPE is particularly sensitive to near-zero observed power. The following analysis further compares multi-step forecasting performance against the other benchmark models. Table 10 reports the four metrics on all datasets.

On Dataset 1, CNN–iTransformer–LSTM reduces LSTM RMSE by 31.69%, 23.38%, and 11.89% at one, two, and three steps, respectively; MAE falls by 23.53%, 18.60%, and 7.09%. MAPE is 3.04% higher at one step but is lower by 32.31% and 31.66% at the longer horizons. Thus, the benefit of combining complementary modules persists as the forecast step expands.

On Dataset 2, CNN–iTransformer–LSTM lowers CNN RMSE by 0.07%, 8.36%, and 26.06% across the three horizons. The corresponding MAE reductions are 6.09%, 16.98%, and 31.78%, and MAPE decreases by 36.68%, 48.24%, and 41.51%. Compared with LSTM, RMSE is also lower by 9.34%, 12.99%, and 18.30%. These results indicate that the hybrid model proposed in this study retains its forecasting advantage across multi-step prediction.

The effect of decomposition is evident on Dataset 3. Relative to LSTM, VMD–LSTM decreases RMSE by 18.54%, 10.83%, and 28.33% and MAE by 13.14%, 6.94%, and 27.01% for one-, two-, and three-step forecasts.

For Dataset 4, the proposed framework is compared directly with VMD–CNN–iTransformer–LSTM. RMSE decreases by 52.96%, 25.50%, and 15.08% from one to three steps. MAE decreases by 56.56%, 32.11%, and 19.88%. MAPE decreases by 64.81%, 82.70%, and 49.52%. R^2^ remains high at 0.9984, 0.9929, and 0.9893, respectively.

**Figure 17:**
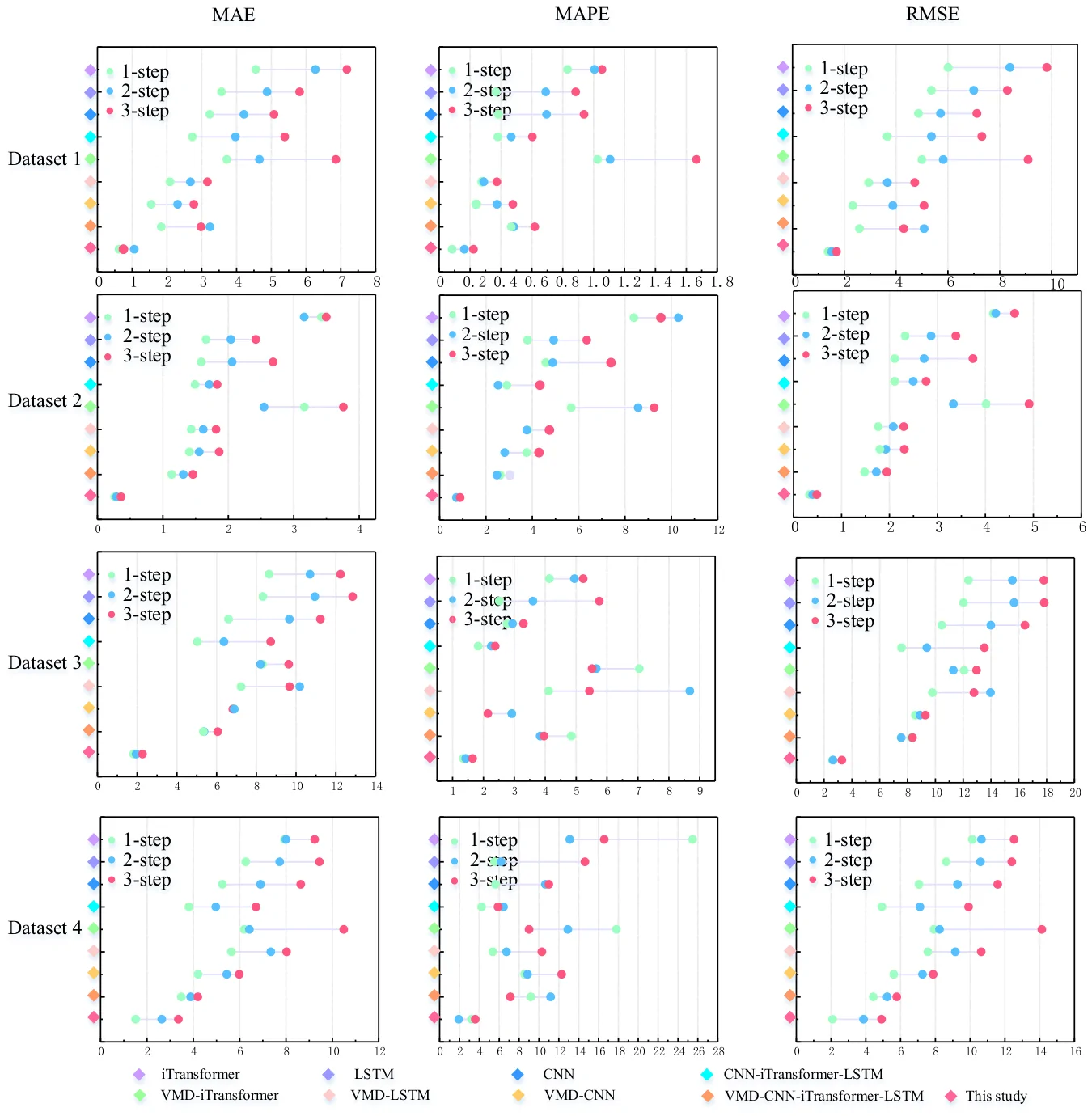
Multi-step prediction model evaluation metrics.

Errors increase with step for most models, as expected. Nevertheless, the proposed framework retains the lowest error levels across the reported multi-step comparisons on all four datasets. Figure 17 visualizes this error metrics.

#### 5.3.3. Comparison of Multiple Optimization Algorithms

In this experiment, HOA was compared with SSA [55], PSO [56], and GWO [57] on Datasets 1 and 3. Each algorithm optimized the VMD–CNN parameters under the same objective, search budget, bounds, and experimental conditions.

**Figure 18:**
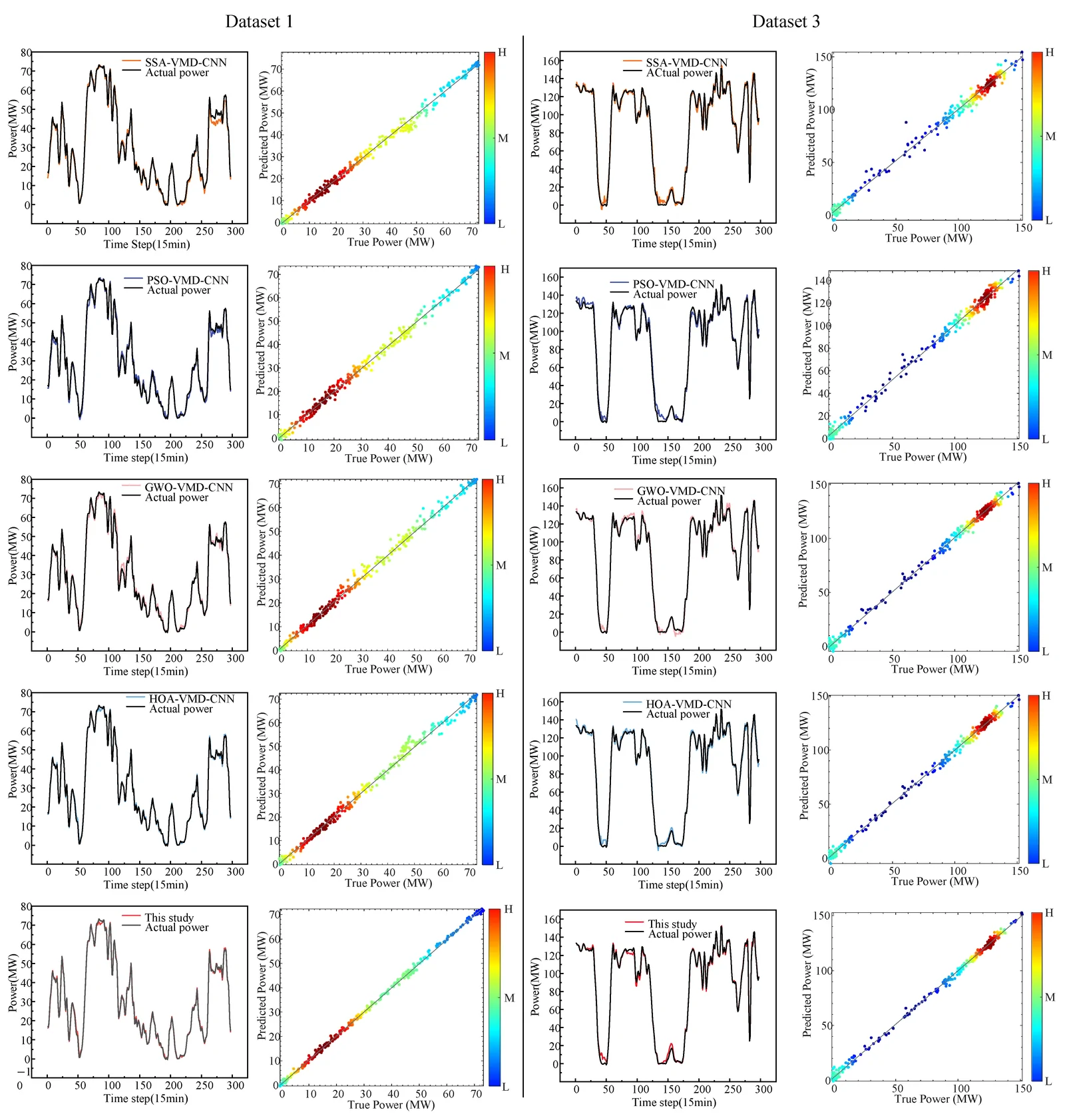
Plot of predicted curves, true versus predicted values, for Datasets 1 and 3.

**Figure 19:**
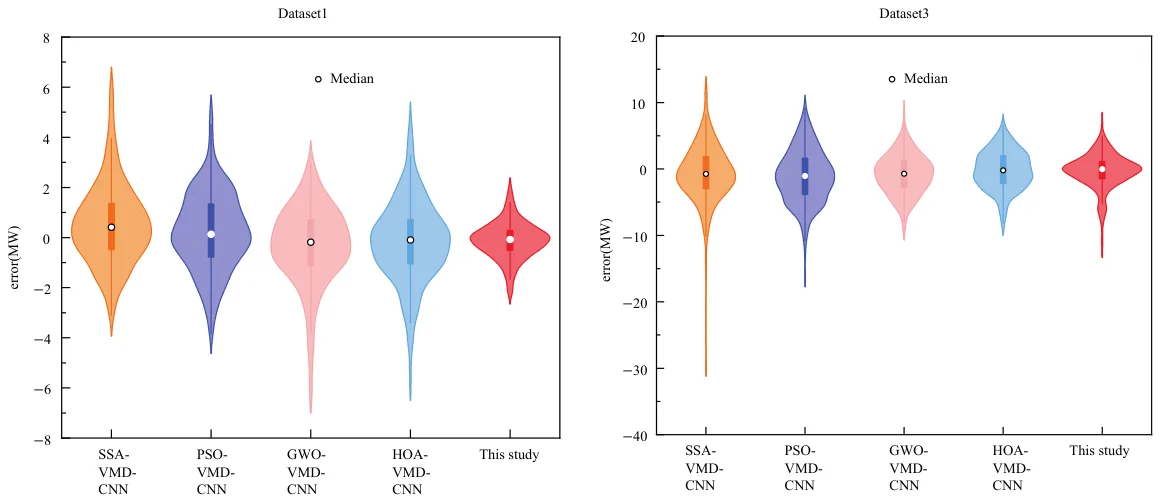
Violin plots for prediction errors for Datasets 1 and 3.

The complete HOA–VMD–CNN–iTransformer–LSTM framework was included to distinguish gains from the optimizer and from the additional iTransformer and LSTM modules. Table 11 reports the evaluation metrics, Figure 18 compares trajectories and observed-versus-predicted scatter, and Figure 19 shows the corresponding error distributions.

(1) For Dataset 1, HOA–VMD–CNN lowers MAE and RMSE by 0.26% and 16.62% relative to GWO–VMD–CNN, indicating that HOA is particularly effective at limiting large deviations on this dataset. Adding iTransformer and LSTM to the HOA model produces further MAE, MAPE, and RMSE reductions of 53.30%, 64.47%, and 42.39%, respectively.

The curves in Figure 18 show that all optimized models follow the broad Dataset 1 trajectory, but the complete framework more closely reproduces rapid changes and peak values. Its points lie nearer the ideal diagonal, and Figure 19 shows a narrower error distribution centered near zero.

(2) For Dataset 3, HOA–VMD–CNN reduced MAE and RMSE by 2.85% and 6.65%, respectively, relative to GWO–VMD–CNN. Its MAPE increased by 44.83%. Near-zero observations in Dataset 3 may increase MAPE. However, the lower MAE suggests that HOA–VMD–CNN generally produces smaller absolute forecasting errors.

The HOA–VMD–CNN–iTransformer–LSTM model achieved MAE, MAPE, and RMSE values of 1.8223, 1.3529, and 2.5964, respectively. Relative to HOA–VMD–CNN, these metrics decreased by 23.22%, 6.50%, and 11.02%, respectively. Its MAE and RMSE were the lowest among the five models. Figure 18 and Figure 19 corroborate these comparisons: HOA–VMD–CNN tracked most power variations, whereas the proposed model followed rapid fluctuations more closely. The error violin plot in Figure 19 further indicates that the prediction errors of the proposed model are mainly distributed around zero, and the overall error distribution is more compact. This is consistent with the analysis of the evaluation metrics and demonstrates that the proposed model has better overall prediction performance.

#### 5.3.4. Analysis of the Time Complexity of the Model

The offline computational procedure consists of three stages: HOA-based VMD optimization and decomposition, HOA-based network hyperparameter optimization, and final network training. The computational cost of each stage is described below.

(1) HOA-based VMD optimization and decomposition. HOA searches for the VMD mode number and penalty factor, using 10 agents and a maximum of 30 iterations. This process takes approximately 10 min.
(2) HOA-based network hyperparameter optimization. A separate HOA search, also using 10 agents and a maximum of 30 iterations, selects the network hyperparameters. This stage takes approximately 3 h.
(3) Final network training. Following hyperparameter selection, the forecasting network is trained using Adam, a batch size of 50, and a maximum of 70 epochs. Validation-based early stopping is not enabled. Table 12 summarizes the recorded training times of the proposed model across the four datasets. For comparison, Table 13 reports the network training times of the benchmark models on Dataset 1, including standalone networks, hybrid architectures, and VMD-based models.

The framework involves repeated model fitting during HOA optimization and separate modeling of decomposed components, adding to its offline computational workload. Future work will investigate more efficient optimization strategies and lightweight network architectures to reduce this workload while maintaining predictive performance.

## 6. Conclusions

This study introduced a Pearson–HOA–VMD–CNN–iTransformer–LSTM framework for short-term WPF. Pearson correlation analysis selected informative meteorological variables, while HOA optimized the VMD parameters and key hyperparameters of the forecasting network. VMD decomposed the original wind power sequence into smoother modal components, reducing the adverse effects of volatility and non-stationarity. At the 15-min forecast horizon, the proposed framework achieved RMSE values of 1.3779, 0.3358, 2.5964, and 2.0852 MW on Datasets 1–4, respectively, with corresponding R^2^ values of 0.9957, 0.9984, 0.9972, and 0.9984. Averaged across the four datasets at this horizon, compared with CNN–iTransformer–LSTM, it achieved average relative reductions of 67.48%, 71.17%, and 49.66% in RMSE, MAE, and MAPE, respectively. Compared with VMD–CNN–iTransformer–LSTM, it achieved average relative reductions of 61.43%, 66.30%, and 72.82% in RMSE, MAE, and MAPE, respectively. Meanwhile, the mean R^2^ increased from 0.9788 to 0.9974. The multi-step forecasting experiments further showed that the proposed framework maintained consistently strong performance across one step to three steps. These results indicate that the combined feature selection and parameter optimization strategies improve forecasting accuracy across the four evaluated datasets.

Despite its improved forecasting performance, the proposed model retains limitations. Although the data were split chronologically, applying VMD to complete validation and test segments may introduce future information into the modal components used for forecasting. This look-ahead information may lead to optimistic estimates of forecasting accuracy. The evaluation covers four one-month subsets from two Chinese wind farms during a single year, with short test periods and single-run results. Robustness to random initialization and generalization to other years, regions, and farms therefore remain unverified. The findings do not establish applicability across offshore, mountainous, or flat continental environments or under extreme weather conditions. The inputs include historical meteorological measurements rather than numerical weather predictions. The outputs are limited to point forecasts at horizons of 45 min or less. The framework does not quantify predictive uncertainty or provide prediction intervals. The iterative HOA search and complex structures increase training time and computational cost. Moreover, although the framework was evaluated using four datasets and multi-step forecasting experiment, its robustness under extreme weather and new wind farms remains to be investigated. Future work should explore lightweight optimization methods and network architectures, probabilistic forecasting, longer forecast horizons, model transferability to new wind farms and validate on open-access international wind power benchmarks and assess generalization across different wind regimes and terrain types.

## Figures and Tables

**Table 1:** Wind power statistics information.

Data	Maximum (MW)	Minimum (MW)	Mean (MW)	Standard Deviation	Capacity Factor (%)	Zero Samples (%)
Dataset 1	79.8506	0.0675	27.2088	24.4873	27.4836	0.0000
Dataset 2	91.9480	0.0526	19.9543	24.7917	20.1559	0.0000
Dataset 3	192.8377	0.0000	95.2670	47.7680	47.6335	3.3602
Dataset 4	171.1810	0.0000	81.9085	47.9360	40.9543	5.0067

**Table 2:** Pearson correlation coefficients between meteorological variables and wind power for Datasets 1 and 2.

Dataset 1	Correlation Coefficient	Dataset 2	Correlation Coefficient
Wind speed at hub height	0.78631	Wind speed at 50 m	0.75935
Wind speed at 50 m	0.77696	Wind speed at hub height	0.75807
Wind speed at 30 m	0.74929	Wind speed at 30 m	0.73888
Wind speed at 10 m	0.67021	Wind speed at 10 m	0.67733
Wind direction at 50 m	0.41784	Wind direction at 50 m	0.38179
Wind direction at hub height	0.41744	Wind direction at hub height	0.36377
Wind direction at 30 m	0.39461	Wind direction at 30 m	0.32713
Wind direction at 10 m	0.29660	Wind direction at 10 m	0.29520
Relative humidity	0.28075	Relative humidity	0.12180
Air temperature	0.02670	Air temperature	0.05883
Atmospheric pressure	−0.00621	Atmospheric pressure	0.00842

**Table 3:** Pearson correlation coefficients between meteorological variables and wind power for Datasets 3 and 4.

Dataset 3	Correlation Coefficient	Dataset 4	Correlation Coefficient
Wind speed at hub height	0.78485	Wind speed at hub height	0.81387
Wind speed at 50 m	0.77764	Wind speed at 50 m	0.80737
Wind speed at 30 m	0.76399	Wind speed at 30 m	0.79929
Wind speed at 10 m	0.73762	Wind speed at 10 m	0.78422
Wind direction at 10 m	0.50712	Wind direction at 10 m	0.54860
Wind direction at 30 m	0.48110	Wind direction at 30 m	0.49515
Wind direction at 50 m	0.46071	Wind direction at 50 m	0.45313
Wind direction at hub height	0.45058	Wind direction at hub height	0.41853
Air temperature	−0.12111	Air temperature	0.023637
Atmospheric pressure	0.095392	Atmospheric pressure	−0.0088909
Relative humidity	0.095339	Relative humidity	−0.0088318

**Table 4:** Optimization results of HOA–VMD.

Parameters	Dataset 1	Dataset 2	Dataset 3	Dataset 4
*k*	9	9	7	7
α	1085	1106	586	731

**Table 5:** Optimal hyperparameters for HOA–CNN–iTransformer–LSTM.

Hyperparameters	Dataset 1	Dataset 2	Dataset 3	Dataset 4
Position-encoding dimension	8	64	8	64
Number of attention heads	16	32	4	64
Number of attention keys	32	64	8	128
Number of LSTM neurons	128	64	8	256
Learning rate	0.01	0.01	0.01	0.01
Dropout rate	0.5	0.5	0.5	0.5

**Table 6:** MAE, MAPE, RMSE, and R^2^ for the models used to predict Datasets 1 and 2.

Model	Dataset 1				Dataset 2			
	MAE	MAPE	RMSE	R^2^	MAE	MAPE	RMSE	R^2^
iTransformer	4.5596	1.0545	6.0148	0.9178	3.4251	8.3870	4.1705	0.7478
TimesNet	4.6391	1.0978	6.1800	0.9195	1.4723	4.0826	2.4278	0.9216
PatchTST	3.9255	0.6766	5.4340	0.9377	1.3861	3.9624	2.3605	0.9255
Dlinear	3.5603	0.5412	4.9923	0.9475	1.5179	3.9186	2.3695	0.9249
LSTM	3.5712	0.3698	5.3622	0.9346	1.6606	3.7969	2.3294	0.9213
CNN	3.2308	0.3837	4.8651	0.9462	1.5889	4.5846	2.1133	0.9352
CNN–LSTM	5.4469	0.5855	7.4359	0.8743	1.6099	2.7282	2.3809	0.9178
iTransformer–LSTM	4.2154	0.9657	5.6082	0.9285	2.2844	8.3267	2.7880	0.8873
BiLSTM	3.1035	0.3640	4.3485	0.9570	1.6841	4.0900	2.2005	0.9298
CNN–iTransformer–LSTM	2.7309	0.3799	3.6627	0.9695	1.4921	2.9030	2.1119	0.9353
VMD–iTransformer	4.6637	1.0250	5.8224	0.9230	3.1633	9.2665	4.0199	0.7657
VMD–LSTM	2.0895	0.2794	2.9438	0.9803	1.4339	3.8067	1.7675	0.9547
VMD–CNN	1.5501	0.2417	2.3333	0.9876	1.5566	3.7644	1.9262	0.9462
VMD–CNN–iTransformer–LSTM	1.8369	0.6195	2.5888	0.9848	1.1347	2.6234	1.4828	0.9681
HOA–VMD–CNN–iTransformer–LSTM	1.6634	0.6046	2.3671	0.9873	1.1396	2.0797	1.3862	0.9721
VMD–HOA–CNN–iTransformer–LSTM	1.0130	0.1508	1.3966	0.9956	0.5241	0.7931	0.7452	0.9919
This study	0.6255	0.0848	1.3779	0.9957	0.2650	0.8372	0.3358	0.9984

**Table 7:** MAE, MAPE, RMSE, and R2 for the models used to predict Datasets 3 and 4.

Model	Dataset 3				Dataset 4			
	MAE	MAPE	RMSE	R^2^	MAE	MAPE	RMSE	R^2^
iTransformer	8.6456	4.1405	12.3893	0.9368	7.9421	25.4886	10.1358	0.9491
TimesNet	9.5151	4.7163	13.9147	0.9221	6.0317	5.6082	8.7695	0.9609
PatchTST	7.9085	2.3748	11.3831	0.9479	5.9362	5.5896	8.8189	0.9604
Dlinear	8.0407	2.4627	11.8818	0.9432	5.8623	5.5637	8.2737	0.9652
LSTM	8.3306	2.5142	12.0268	0.9404	6.2680	5.5423	8.6289	0.9631
CNN	6.6002	2.1360	10.4734	0.9548	5.2601	5.6360	7.0656	0.9753
CNN–LSTM	9.0915	1.7203	13.8162	0.9214	6.9938	4.6332	10.0019	0.9504
iTransformer–LSTM	6.3470	3.3200	9.1818	0.9653	5.0338	7.4354	6.8585	0.9767
BiLSTM	7.2399	1.9626	10.7391	0.9525	5.5248	4.9696	7.5852	0.9715
CNN–iTransformer–LSTM	5.2084	1.8322	7.5799	0.9763	3.8162	4.2339	4.9286	0.9879
VMD–iTransformer	8.3101	7.0458	12.0759	0.9399	6.2072	17.8136	7.9252	0.9689
VMD–LSTM	7.2362	4.1109	9.7966	0.9605	5.6455	5.3566	7.5696	0.9731
VMD–CNN	6.9015	2.9244	8.5754	0.9697	4.2084	8.5716	5.6112	0.9844
VMD–CNN–iTransformer–LSTM	5.3666	4.8472	8.2814	0.9718	3.4844	9.1881	4.4331	0.9903
HOA–VMD–CNN–iTransformer–LSTM	4.1263	2.5542	5.4487	0.9878	3.2101	7.2327	4.0020	0.9921
VMD–HOA–CNN–iTransformer–LSTM	2.4237	1.4599	3.7636	0.9942	1.6173	3.4665	2.2318	0.9975
This study	1.8223	1.3529	2.5964	0.9972	1.5137	3.2337	2.0852	0.9984

**Table 8:** Training, validation, and test MAE of the proposed model across the four datasets.

Dataset	Training MAE	Validation MAE	Test MAE
Dataset 1	0.43484	0.44470	0.62555
Dataset 2	0.50991	0.27706	0.26504
Dataset 3	0.85850	2.48247	1.82226
Dataset 4	1.54240	1.14260	1.5137

**Table 9:** Comparison of the proposed model and persistence baseline.

Dataset	Model	1-STEP				2-STEP				3-STEP			
		MAE	MAPE	RMSE	R^2^	MAE	MAPE	RMSE	R^2^	MAE	MAPE	RMSE	R^2^
Dataset 1	Persistence baseline	2.8589	0.2462	4.0136	0.9634	4.0351	0.3965	5.8854	0.9213	5.0028	0.5569	7.3313	0.8779
Dataset 2	Persistence baseline	1.0013	0.6977	1.6142	0.9622	1.4280	1.2747	2.4105	0.9128	1.7818	1.9671	3.0641	0.8642
Dataset 3	Persistence baseline	5.3706	0.3403	8.5060	0.9702	7.7012	0.4999	12.6648	0.9340	9.7158	0.6715	16.0614	0.8940
Dataset 4	Persistence baseline	4.1256	0.5352	6.1736	0.9811	5.8798	0.7019	9.1417	0.9588	7.3869	0.8216	11.5493	0.9344
Dataset 1	This study	0.6255	0.0848	1.3779	0.9957	0.7533	0.1643	1.5204	0.9949	1.0608	0.2224	1.7018	0.9937
Dataset 2	This study	0.2650	0.8372	0.3358	0.9984	0.2921	0.7291	0.4028	0.9979	0.3619	0.8913	0.4873	0.9971
Dataset 3	This study	1.8223	1.3529	2.5964	0.9972	1.9559	1.4300	2.6520	0.9973	2.2728	1.6537	3.2831	0.9961
Dataset 4	This study	1.5137	3.2337	2.0852	0.9984	2.6409	1.9352	3.8951	0.9929	3.3611	3.6033	4.9125	0.9893

**Table 10:** Multi-step prediction evaluation metrics.

Dataset	Model	1-STEP				2-STEP				3-STEP			
		MAE	MAPE	RMSE	R2	MAE	MAPE	RMSE	R2	MAE	MAPE	RMSE	R2
Dataset 1	iTransformer	4.5596	1.0545	6.0148	0.9178	6.2699	0.8319	8.3968	0.8466	7.1774	1.0059	9.8197	0.7981
	LSTM	3.5712	0.3698	5.3622	0.9346	4.8808	0.6904	7.0024	0.8925	5.8021	0.8841	8.3027	0.8504
	CNN	3.2308	0.3837	4.8651	0.9462	4.2176	0.6959	5.7177	0.9302	5.0824	0.9378	7.1239	0.8927
	CNN–iTransformer–LSTM	2.7309	0.3799	3.6627	0.9695	3.9729	0.4673	5.3655	0.9373	5.3906	0.6042	7.3152	0.8853
	VMD–iTransformer	4.6637	1.0250	5.8224	0.9230	3.7253	1.1071	5.0002	0.9472	6.8633	1.6662	9.1005	0.8258
	VMD–LSTM	2.0895	0.2794	2.9438	0.9803	2.6761	0.2900	3.6670	0.9702	3.1668	0.3744	4.7249	0.9527
	VMD–CNN	1.5501	0.2417	2.3333	0.9876	2.3078	0.3705	3.8769	0.9787	2.7743	0.4777	5.0814	0.9479
	VMD–CNN–iTransformer–LSTM	1.8369	0.6195	2.5888	0.9848	3.2393	0.4831	5.0855	0.9425	2.9799	0.4674	4.2969	0.9637
	This study	0.6255	0.0848	1.3779	0.9957	0.7533	0.1643	1.5204	0.9949	1.0608	0.2224	1.7018	0.9937
Dataset 2	iTransformer	3.4251	8.3870	4.1705	0.7478	3.1604	10.3083	4.2191	0.7636	3.4983	9.5539	4.6132	0.7414
	LSTM	1.6606	3.7969	2.3294	0.9213	2.0415	4.9267	2.8698	0.8857	2.4228	6.3527	3.3872	0.8463
	CNN	1.5889	4.5846	2.1133	0.9352	2.0610	4.8889	2.7249	0.9042	2.6854	7.4052	3.7427	0.8215
	CNN–iTransformer–LSTM	1.4921	2.9030	2.1119	0.9353	1.7111	2.5306	2.4971	0.9161	1.8320	4.3311	2.7672	0.9002
	VMD–iTransformer	3.1633	9.2665	4.0199	0.7657	2.5463	5.6783	3.3346	0.8685	3.7606	8.5709	4.9131	0.7126
	VMD–LSTM	1.4339	3.8067	1.7675	0.9547	1.6180	3.7646	2.0827	0.9419	1.8148	4.7426	2.3009	0.9322
	VMD–CNN	1.5566	3.7644	1.9262	0.9462	1.4075	2.8130	1.8304	0.9614	1.8616	4.2962	2.3119	0.9542
	VMD–CNN–iTransformer–LSTM	1.1347	2.6234	1.4828	0.9681	1.3141	2.4793	1.7287	0.9587	1.4618	3.0432	1.9487	0.9511
	This study	0.2650	0.8372	0.3358	0.9984	0.2921	0.7291	0.4028	0.9979	0.3619	0.8913	0.4873	0.9971
Dataset 3	iTransformer	8.6456	4.1405	12.3893	0.9368	10.7088	4.9486	15.5520	0.9052	12.2415	5.2337	17.8156	0.8812
	LSTM	8.3306	2.5142	12.0268	0.9404	10.9502	3.6062	15.6698	0.9022	12.8515	5.7547	17.8363	0.8785
	CNN	6.6002	2.1360	10.4734	0.9548	9.6626	3.2996	14.0002	0.9249	11.2263	2.9477	16.4553	0.8981
	CNN–iTransformer–LSTM	5.2084	1.8322	7.5799	0.9763	6.3708	2.2573	9.3944	0.9656	8.7249	2.3836	13.5348	0.9294
	VMD–iTransformer	8.3101	7.0458	12.0759	0.9399	8.2105	5.6543	11.2975	0.9516	9.6388	5.5179	12.9703	0.9294
	VMD–LSTM	7.2362	4.1109	9.7966	0.9605	10.1908	8.6825	13.9731	0.9278	9.3806	5.4331	12.7826	0.9398
	VMD–CNN	6.9015	2.9244	8.5754	0.9697	6.8197	2.9221	8.8923	0.9700	6.8876	2.1465	9.2802	0.9717
	VMD–CNN–iTransformer–LSTM	5.3666	4.8472	8.2814	0.9718	5.3398	3.8424	7.5494	0.9783	6.0553	3.9620	8.3621	0.9751
	This study	1.8223	1.3529	2.5964	0.9972	1.9559	1.4300	2.6520	0.9973	2.2728	1.6537	3.2831	0.9961
Dataset 4	iTransformer	7.9421	25.4886	10.1358	0.9491	7.9427	13.1141	10.6562	0.9476	9.2446	16.5796	12.5349	0.9341
	LSTM	6.2680	5.5423	8.6289	0.9631	7.7303	6.1902	10.6038	0.9468	9.4433	14.6412	12.4010	0.9303
	CNN	5.2601	5.6360	7.0656	0.9753	6.9067	10.6582	9.2784	0.9613	8.6423	11.0011	11.5904	0.9429
	CNN–iTransformer–LSTM	3.8162	4.2339	4.9286	0.9879	4.9721	6.4504	7.1225	0.9761	6.7106	5.8886	9.9134	0.9549
	VMD–iTransformer	6.2072	17.8136	7.9252	0.9689	6.4260	12.9214	8.2461	0.9695	10.4942	9.0189	14.1290	0.9342
	VMD–LSTM	5.6455	5.3566	7.5696	0.9731	7.3496	21.7419	9.1586	0.9612	8.0289	10.2994	10.6431	0.9487
	VMD–CNN	4.2084	8.5716	5.6112	0.9844	5.9856	12.2840	7.8761	0.9728	5.4430	8.8588	7.2689	0.9780
	VMD–CNN–iTransformer–LSTM	3.4844	9.1881	4.4331	0.9903	3.8897	11.1872	5.2284	0.9877	4.1951	7.138	5.7848	0.9859
	This study	1.5137	3.2337	2.0852	0.9984	2.6409	1.9352	3.8951	0.9929	3.3611	3.6033	4.9125	0.9893

**Table 11:** Model prediction results optimized using multiple optimization algorithms.

Model	Dataset 1 MAE	MAPE	RMSE	Dataset 3 MAE	MAPE	RMSE
SSA–VMD–CNN	1.3856	0.1999	2.5933	3.1845	1.4608	4.3652
PSO–VMD–CNN	1.3489	0.2493	2.6167	3.3285	2.0492	4.1668
GWO–VMD–CNN	1.3429	0.1721	2.8685	2.4430	0.9990	3.1260
HOA–VMD–CNN	1.3394	0.2387	2.3918	2.3734	1.4469	2.9180
This study	0.6255	0.0848	1.3779	1.8223	1.3529	2.5964

**Table 12:** Training times of the proposed model.

Dataset	Training Time(s)
Dataset 1	1727
Dataset 2	1574
Dataset 3	1432
Dataset 4	1648

**Table 13:** Training times of the benchmarks model for Dataset 1.

Model	Training Time(s)
iTransformer	9
CNN	16
LSTM	7
BiLSTM	6
CNN–LSTM	17
iTransformer–LSTM	8
CNN–iTransformer–LSTM	10
VMD–iTransformer	27
VMD–CNN	64
VMD–LSTM	66

## Data Availability

The data that support the findings of this study are contained within the article.
